# TPM1 mediates inflammation downstream of TREM2 via the PKA/CREB signaling pathway

**DOI:** 10.1186/s12974-022-02619-3

**Published:** 2022-10-14

**Authors:** Rong Li, Jing Zhang, Qiong Wang, Meng Cheng, Bin Lin

**Affiliations:** 1 School of Optometry, The Hong Kong Polytechnic University, Hung Hom, Kowloon, Hong Kong; 2 Centre for Eye and Vision Research (CEVR), 17W Hong Kong Science Park, Shatin, Hong Kong; 3 Research Centre for SHARP Vision (RCSV), The Hong Kong Polytechnic University, Kowloon, Hong Kong

**Keywords:** Retina, Tropomyosin 1, Inflammation, TREM2, CREB

## Abstract

**Background:**

Microglia, the innate immune cells in the central nervous system, play an essential role in brain homeostasis, neuroinflammation and brain infections. Dysregulated microglia, on the other hand, are associated with neurodegenerative diseases, yet the mechanisms underlying pro-inflammatory gene expression in microglia are incompletely understood.

**Methods:**

We investigated the role of the actin-associated protein tropomyosin 1 (TPM1) in regulating pro-inflammatory phenotype of microglia in the retina by using a combination of cell culture, immunocytochemistry, Western blot, qPCR, TUNEL, RNA sequencing and electroretinogram analysis. TREM2^−/−^ mice were used to investigate whether TPM1 regulated pro-inflammatory responses downstream of TREM2. To conditionally deplete microglia, we backcrossed CX3CR1^CreER^ mice with Rosa26^iDTR^ mice to generate CX3CR1^CreER^:Rosa26^iDTR^ mice.

**Results:**

We revealed a vital role for TPM1 in regulating pro-inflammatory phenotype of microglia. We found that TPM1 drove LPS-induced inflammation and neuronal death in the retina via the PKA/CREB pathway. TPM1 knockdown ameliorated LPS-induced inflammation in WT retinas yet exaggerated the inflammation in TREM2^−/−^ retinas. RNA sequencing revealed that genes associated with M1 microglia and A1 astrocytes were significantly downregulated in LPS-treated WT retinas but upregulated in LPS-treated TREM2^−/−^ retinas after TPM1 knockdown. Mechanistically, we demonstrated that CREB activated by TPM1 knockdown mediated anti-inflammatory genes in LPS-treated WT retinas but pro-inflammatory genes in LPS-treated TREM2^−/−^ retinas, suggesting a novel role for TREM2 as a brake on TPM1-mediated inflammation. Furthermore, we identified that TPM1 regulated inflammation downstream of TREM2 and in a microglia-dependent manner.

**Conclusions:**

We demonstrate that TPM1 mediates inflammation downstream of TREM2 via the PKA/CREB signaling pathway. Our findings suggest that TPM1 could be a potential target for therapeutic intervention in brain diseases.

**Supplementary Information:**

The online version contains supplementary material available at 10.1186/s12974-022-02619-3.

## Introduction

Microglia, the principal resident immune cells of the central nervous system (CNS), serve as sensors and executers of innate immunity. Increasing evidence indicates that dysregulated microglia is associated with various common human neurodegenerative diseases such as Alzheimer’s disease (AD) [1]. It has been previously shown that excessive activation of microglia enhances the production of a variety of cytokines and chemokines and exacerbates neuronal cell death in neurological disorders [2–4]. Therefore, targeting the cellular mechanisms controlling microglial dysregulation may arrest or reverse neurodegenerative diseases. However, the mechanisms underlying pro-inflammatory gene expression in microglia are incompletely understood.

Tropomyosins (TPM) are a family of actin-associated proteins implicated in the pathology of neurodegenerative disease and neurological disorders [5, 6]. TPM1, a widely expressed actin‑binding protein of TPM family, is elevated in brains from AD patients compared to healthy controls [6]. Similarly, we observed that TPM1 protein is more abundant in retinas of young AD mouse models than in retinas of age-matched control mice [7]. We have recently reported that the elevation of systemic TPM1 induces endogenous TPM1 upregulation, inflammation and neuronal remodeling in the aging retina by regulating the fatty acid-binding protein 5/NF-κB and protein kinase A (PKA) signaling pathways [7]. However, how endogenous TPM1 triggers inflammation and neuronal death in the retina is unclear. Here, we investigated the role of endogenous TPM1 in regulating pro-inflammatory gene expression in microglia in the retina. To determine the specific role of microglia in TPM1-mediated inflammatory processes, we used CX3CR1^CreER/+^:R26^iDTR/+^ mice for microglial ablation from the CNS including the retina. We demonstrated that TPM1 was involved in LPS-induced inflammation, and it drove LPS-induced glial reaction, the production of pro-inflammatory cytokines, neuronal apoptosis and functional decline in the C57BL/6J mouse (WT) retina through PKA and its downstream effector cAMP-responsive element binding protein (CREB). In the absence of microglia, however, TPM1 knockdown failed to suppress LPS-induced inflammation in the WT retina, confirming that TPM1 regulated LPS-induced inflammation in a microglia-dependent manner.

Triggering receptor expressed on myeloid cells 2 (TREM2) is specifically expressed by microglia in the brain [8]. As a microglial surface receptor, TREM2 interacts with the signal transduction partner DAP12 to initiate signal transduction pathways that promote microglial cell activation and phagocytosis [9]. TREM2 variants are reported to be associated with common neurodegenerative diseases, including Alzheimer’s disease (AD) [10–12]. In addition, TREM2 is previously reported to modulate toll-like receptor 4 (TLR4) and TLR4-mediated inflammation [13, 14]. Therefore, we investigated whether TPM1 regulated inflammation via the TREM2/TLR4 pathway. Surprisingly, we found that TPM1 knockdown ameliorated LPS-induced inflammation in the WT retina but markedly aggravated LPS-induced inflammation, neuronal death and function decline in the TREM2^−/−^ mouse retina. RNA sequencing and bioinformatics analysis revealed that TPM1 altered gene signatures in glial cells including microglia and astrocytes, and regulated genes that were associated with inflammation-related pathways in TREM2^−/−^ retinas. Interestingly, we observed that CREB activated by TPM1 knockdown promoted production of anti-inflammatory genes, such as IL-10, in WT retinas but increased production of pro-inflammatory cytokines, such as IL-6, in TREM2^−/−^ retinas, indicating a novel role for TREM2 serving as a brake on TPM1-mediated inflammation. Moreover, we identified that TPM1 mediated inflammation downstream of the TREM2 signal and via TLR4 signal. Collectively, our results demonstrate that TPM1 is an important regulator of pro-inflammatory gene expression in microglia and that TPM1 could be a potential target for therapeutic intervention in brain diseases.

## Results

### TPM1 regulates inflammation via the PKA/CREB pathway in vitro

**Fig. 1:**
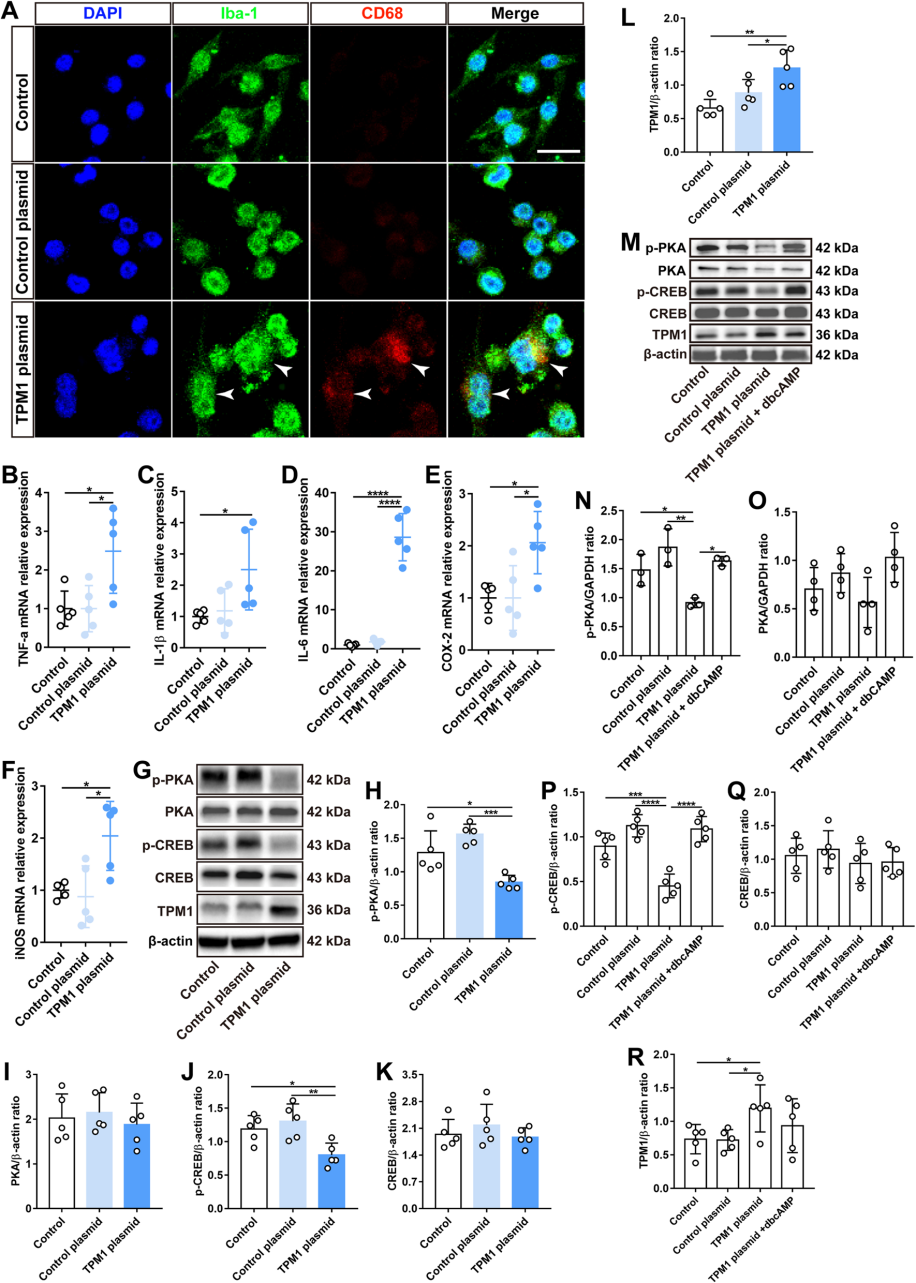
TPM1 induces inflammation by regulating the PKA/CREB pathway in BV2 cells. **A** Immunostaining of BV2 cells with antibodies against Iba-1 and CD68 after treatment with TPM1 plasmid or control plasmid. Arrowheads show the colocalization of Iba-1-positive microglial cells with CD68 signal. Scale bar, 20 µm. **B**–**F** qPCR analysis of TNF-α, IL-1β, IL-6, COX-2 and iNOS in BV2 cells treated with TPM1 plasmid or control plasmid. Data are presented as mean ± SEM and analyzed by one-way ANOVA with Tukey’s multiple comparison test (compared to TPM1 plasmid, *p < 0.05, ****p < 0.001). Five independent experiments were performed. **G**–**L** Western blot analysis (**G**) and quantification of p-PKA, PKA, p-CREB, CREB and TPM1 (**H**–**L**) in BV2 cells following TPM1 plasmid or control plasmid treatment. Data are presented as mean ± SEM and analyzed by one-way ANOVA with Tukey’s multiple comparison test (compared to TPM1 plasmid, *p < 0.05, **p < 0.01, ***p < 0.001). Five independent experiments were performed. **M**–**R** Western blot analysis (**M**) and quantification of p-PKA, PKA, p-CREB, CREB and TPM1 (**N**–**R**) in BV2 cells treated by TPM1 plasmid or control plasmid and followed by dbcAMP treatment. Data are presented as mean ± SEM analyzed by one-way ANOVA with Tukey’s multiple comparison test (compared to TPM1 plasmid, *p < 0.05, **p < 0.01, ***p < 0.001 ****p < 0.001)

We have recently shown that systemic TPM1 induces endogenous TPM1 upregulation and pro-inflammatory responses in the aging retina [7]. However, it remains unclear how endogenous TPM1 triggers neuroinflammation in the retina. To this end, we transfected BV2 cells, a murine microglia cell line, with TPM1 plasmids (Additional file 1: Fig. S1A). After 24 h incubation, we found that TPM1 overexpression activated microglia by increasing CD68 immunoreactivity in microglia (Fig. 1A; Additional file 1: Fig. S1B, C), and releasing more pro-inflammatory cytokines (TNF-α, IL-1β and IL-6) and chemokines (COX-2 and iNOS) relative to BV2 controls (Fig. 1B–F), suggesting that TPM1 overexpression induces inflammation. In addition, we found that TPM1 overexpression significantly downregulated the phosphorylation of both PKA and CREB in BV2 cells (Fig. 1G–L), suggesting that TPM1 potentially regulates inflammation via the PKA/CREB signaling pathway. Indeed, we observed that TPM1 overexpression failed to downregulate phosphorylated CREB (p-CREB) in BV2 cells after treatment with dbcAMP, an activator of PKA (Fig. 1M–R), confirming that TPM1 mediates neuroinflammation by regulating the PKA/CREB signaling pathway. Consistently, the PKA/CREB signaling pathway is previously reported to regulate neuroinflammation in the CNS [15–17].

To further study the role of TPM1 in regulating neuroinflammation, we treated BV2 cells with lipopolysaccharides (LPS), which is an endotoxin and is responsible for various chronic peripheral neuroinflammation [18]. We found that LPS significantly stimulated endogenous TPM1 expression (Additional file 2: Fig. S2A) and the release of pro-inflammatory cytokines and chemokines in BV2 cells (Additional file 2: Fig. S2B–F), which were then counteracted by TPM1 knockdown using TPM1-specific siRNA (Additional file 1: Fig. S1D–F, Additional file 2: Fig. S2A–F), suggesting that TPM1 is involved in LPS-induced inflammation. Indeed, we found that LPS treatment increased the number of activated microglia with CD68 immunoreactivity, which was then suppressed by TPM1 knockdown (Additional file 1: Fig. S1G, H; Additional file 2: Fig. S2G), confirming the involvement of TPM1 in LPS-mediated inflammation. In addition, we observed that LPS treatment deactivated the PKA/CREB signaling pathway in BV2 cells by downregulating phosphorylation of both PKA and CREB (Additional file 2: Fig. S2H–M). However, the downregulation of p-CREB induced by LPS was reversed following TPM1 knockdown in BV2 cells (Additional file 2: Fig. S2H, K and M), suggesting that TPM1 is involved in LPS-induced neuroinflammation through the regulation of CREB phosphorylation. Furthermore, we found that TPM1 knockdown failed to upregulate p-CREB in LPS-treated BV2 cells after application of H89, an inhibitor of PKA (Additional file 1: Fig. S1I, J; Additional file 2: Fig. S2N–Q), confirming that PKA is involved in TPM1-regulated inflammation. Taken together, these data indicate that TPM1 potentially regulates neuroinflammation via the PKA/CREB signaling pathway.

### TPM1 knockdown reduces LPS-induced inflammation and function decline via the PKA/CREB pathway in vivo

**Fig. 2:**
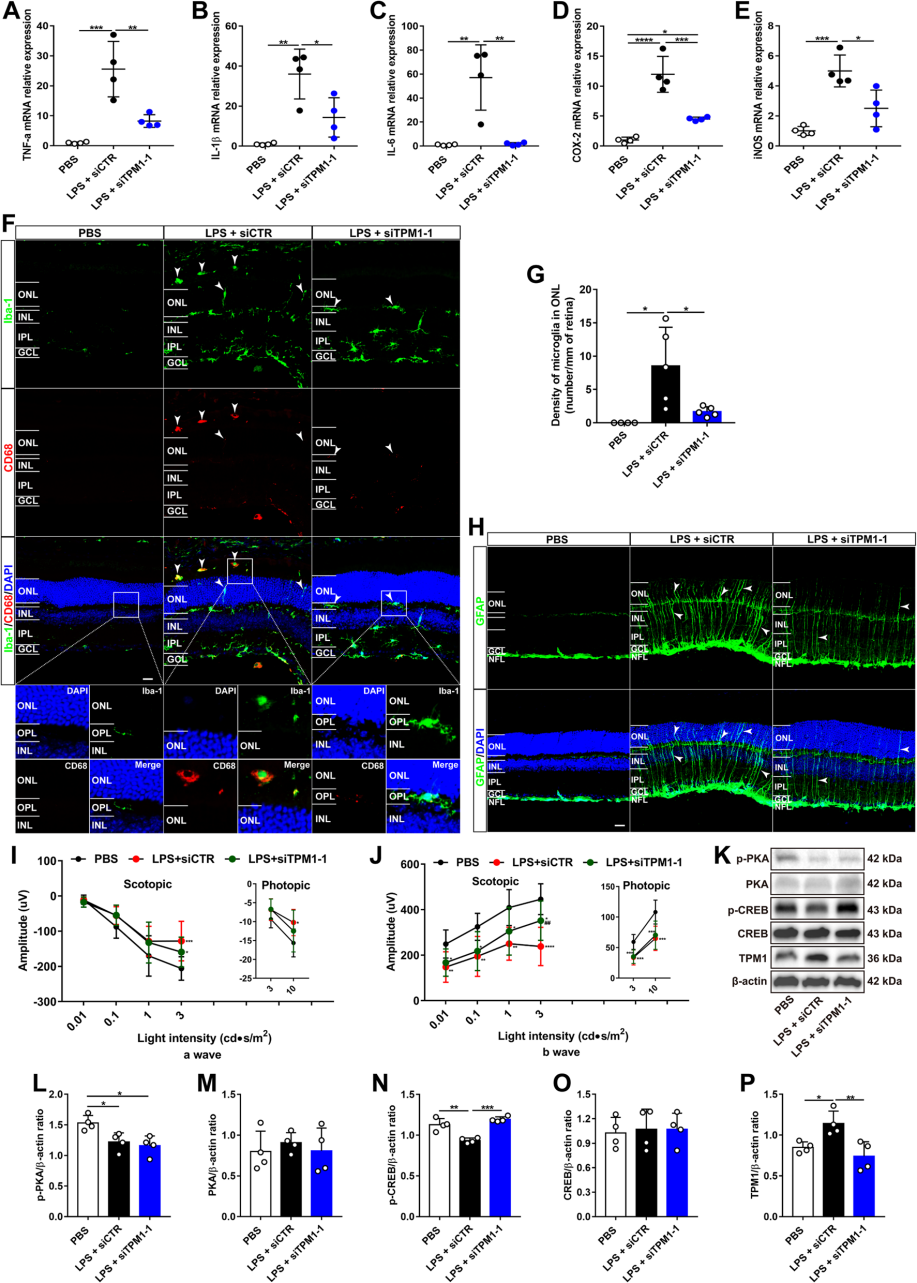
TPM1 knockdown reduces LPS-induced inflammation and function decline via the PKA/CREB pathway in vivo. **A**–**E** qPCR analysis of TNF-α, IL-1β, IL-6, COX-2 and iNOS in C57BL/6J mice after treatments with PBS, or with LPS and siTPM1-1 or siCTR. Data are presented as mean ± SEM and analyzed by one-way ANOVA with Tukey’s multiple comparison test (compared to LPS + siCTR, *p < 0.05, **p < 0.01, ***p < 0.001, ****p < 0.001), *n* = 4 mice in each group. **F**, **G** Representative confocal images of microglia from retinal sections stained with antibodies against Iba-1 and CD68 (**F**) and quantification of microglial cell density in the ONL (**G**) of C57BL/6J mice following treatments with PBS, or with LPS and siTPM1-1 or siCTR. Data are presented as mean ± SEM and analyzed by one-way ANOVA with Tukey’s multiple comparison test (compared to LPS + siCTR, *p < 0.05), *n* = 5 mice in each group. Arrowheads show the migration of activated microglia to the ONL. The boxed regions are highly magnified at the bottom showing migrated microglia in the ONL. Scale bars, 20 µm. **H** Retina sections stained with GFAP antibody. Arrowheads show activated astrocytes and Müller cells. Scale bars, 20 µm. **I**, **J** ERG recording in C57BL/6J mice after treatments with PBS, or with LPS and siTPM1-1 or siCTR. Data are presented as mean ± SEM and analyzed by one-way ANOVA with Tukey’s multiple comparison test (compared to PBS, *p < 0.05, **p < 0.01, ***p < 0.001, ****p < 0.001; LPS + siCTR vs. LPS + siTPM1-1, ^*##*^*p* < 0.01), *n* = 10 mice in each group. **K**–**P** Western blot analysis (**K**) and quantification of p-PKA, PKA, p-CREB, CREB and TPM1 (**L**–**P**) in C57BL/6J mice following treatments with PBS, or with LPS and siTPM1-1 or siCTR. Data are presented as mean ± SEM and analyzed by one-way ANOVA with Tukey’s multiple comparison test (compared to PBS or LPS + siCTR, *p < 0.05, **p < 0.01, ***p < 0.001). *n* = 4 mice in each group

To confirm our in vitro findings, we further investigated the role of TPM1 in vivo. Similarly, we observed that intraperitoneal injection of LPS induced TPM1 upregulation (Additional file 3: Fig. S3A; Fig. 2K, P), and the release of cytokines including TNF-α, IL-1β and IL-6 (Fig. 2A–C) and chemokines including COX-2 and iNOS (Fig. 2D, E) in the retina of C57BL/6J (WT) mice. After TPM1 knockdown by intravitreal administration of siTPM1, however, we found that LPS-induced release of the cytokines and chemokines was reversed in the retina of WT mice (Additional file 3: Fig. S3A, Fig. 2A–E), indicating that TPM1 knockdown counteracts LPS-induced neuroinflammation in vivo. Moreover, we found that TPM1 knockdown significantly increased the expression of anti-inflammatory cytokine IL-10 in LPS-treated WT retinas (Additional file 3: Fig. S3B), further suggesting that TPM1 mediates LPS-induced inflammation. Microglia in the WT mouse retina are positive for Iba-1 but negative for CD68, and their dendrites are restricted within the outer plexiform layer (OPL), the inner plexiform layer (IPL), or the ganglion cell layer (GCL) [3]. However, we found that LPS treatment increased the number of CD68-positive microglia, especially in the IPL of the WT mouse retina (Additional file 3: Fig. S3C–E), and some activated microglia migrated into the ONL from their normal localization within the OPL compared to control retinas (Fig. 2F, G). Interestingly, TPM1 knockdown decreased the incidence of microglia migration into the ONL induced by LPS treatment (Fig. 2F, G), even though the numbers of activated microglia remained unchanged compared to LPS-treated WT retinas (Additional file 3: Fig. S3C–E). Similarly, we observed that LPS treatment increased GFAP immunoreactivity (Fig. 2H, Additional file 3: Fig. S3F), a marker for astrocytes and activated Müller cells, in the retina of WT mice. The dendritic processes of astrocytes in the WT mouse retina are entirely restricted to the nerve fiber layer (NFL) [19]. After LPS treatment, however, we observed that some processes of activated astrocytes extended well beyond their normal strata within the NFL to the IPL (Fig. 2H). However, TPM1 knockdown partially restored the appropriate dendritic localization of astrocytes in the NFL of LPS-treated WT retinas (Fig. 2H, Additional file 3: Fig. S3F). Functionally, we found that LPS treatment significantly decreased the amplitudes of a- and b-waves of electroretinogram (ERG) under both scotopic and photopic conditions compared to WT controls (Fig. 2I, J), even though latency remained unchanged (Additional file 3: Fig. S3G, H). However, TPM1 knockdown partially rescued LPS-induced function declines on WT mice (Fig. 2I, J). Similarly, we found that LPS treatment downregulated p-PKA and p-CREB in WT retinas (Fig. 2K–O), indicating the deactivation of the PKA/CREB signaling pathway. Following TPM1 knockdown (Fig. 2K, P), however, we found that the LPS-induced downregulation of p-CREB was reversed (Fig. 2K, N), suggesting that TPM1 potentially regulates LPS-induced neuroinflammation via the PKA/CREB signaling pathway.

Collectively, we demonstrate that TPM1 is potentially involved in LPS-induced neuroinflammation, and TPM1 knockdown ameliorates glial reactivity, neuroinflammation and function decline in WT retinas.

### TPM1 knockdown exacerbates inflammation in the TREM2^−/−^ mouse retina

**Fig. 3:**
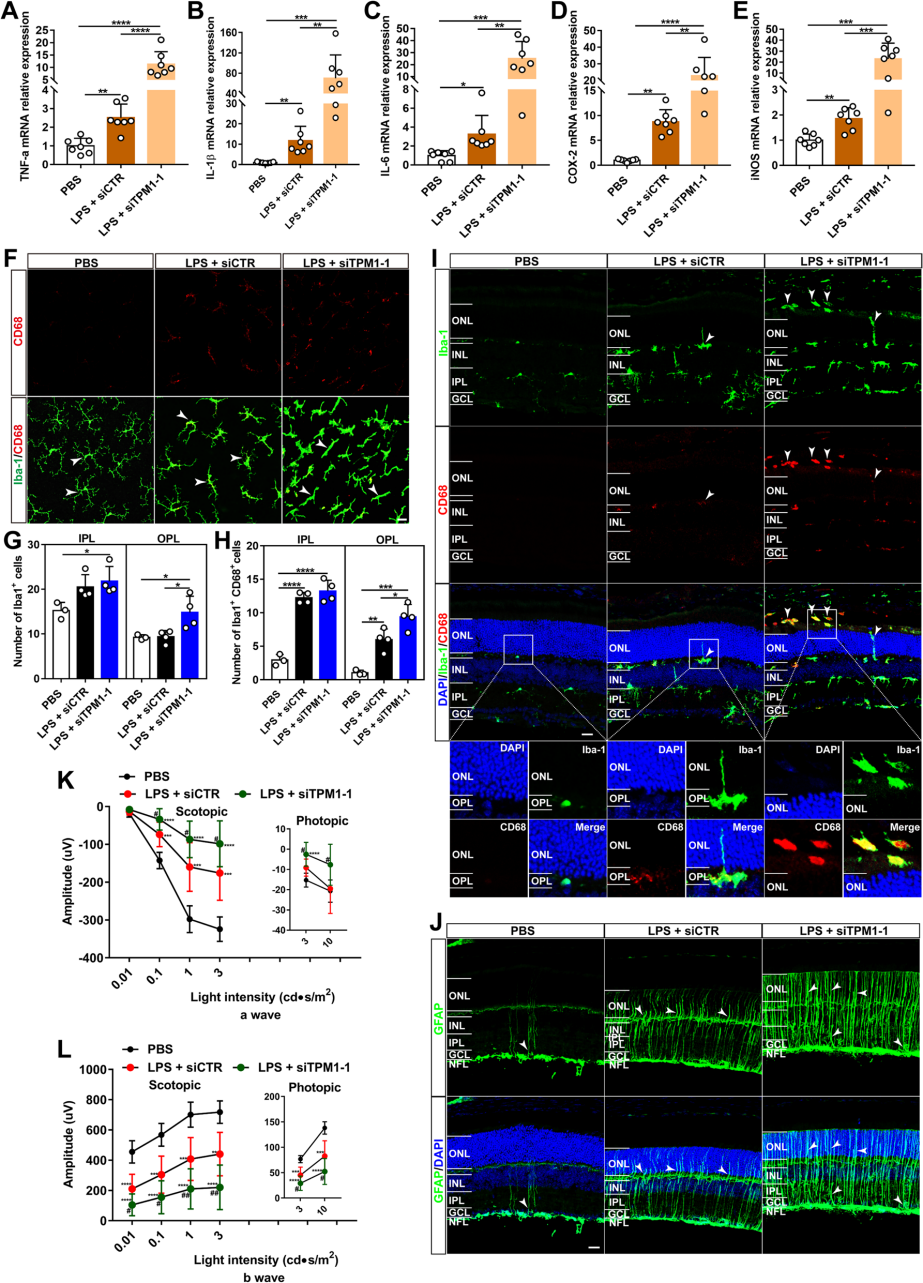
TPM1 knockdown exacerbates inflammation in the TREM2^−/−^ mouse retina. **A**–**E** qPCR analysis of TNF-α, IL-1β, IL-6, COX-2 and iNOS in TREM2^−/−^ mice after treatments with PBS, or with LPS and siTPM1-1 or siCTR. Data are presented as mean ± SEM and analyzed by one-way ANOVA with Tukey’s multiple comparison test (compared to PBS or LPS + siCTR, *p < 0.05, **p < 0.01, ***p < 0.001, ****p < 0.001), *n* = 7 mice in each group. **F**–**H**, Retina whole-mounts stained with antibodies against Iba-1 and CD68 (**F**) and quantification of numbers of Iba-1^+^ (**G**) and of Iba-1^+^CD68^+^ microglia (**H**) in the IPL and OPL of TREM2^−/−^ retinas after treatments with PBS, or with LPS and siTPM1-1 or siCTR. Data are presented as mean ± SEM and analyzed by one-way ANOVA with Tukey’s multiple comparison test (compared to PBS or LPS + siCTR, *p < 0.05, **p < 0.01, ***p < 0.001, ****p < 0.001). *n* = 3 in PBS group, *n* = 4 in LPS + siCTR/siTPM1-1 group. Arrowheads show microglia, scale bar, 20 µm. **I** Retina sections stained with Iba-1 and CD68 antibodies in TREM2^−/−^ retinas following treatments with PBS, or with LPS and siTPM1-1 or siCTR. Arrowheads show the migration of activated microglia to the ONL. The boxed regions are highly magnified at the bottom showing migrated microglia in the ONL. Scale bars, 20 µm. **J** Retina sections stained with an antibody against GFAP in TREM2^−/−^ retinas after treatments with PBS, or with LPS and siTPM1-1 or siCTR. Arrowheads show activated astrocytes and Müller cells. Scale bars, 20 µm. **K**, **L** Scotopic and photopic electroretinogram (ERG) recordings on TREM2^−/−^ mice. Data are presented as mean ± SEM and analyzed by one-way ANOVA with Tukey’s multiple comparison test (compared to PBS, **p < 0.01, ***p < 0.001, ****p < 0.001; LPS + siCTR vs. LPS + siTPM1-1, ^*#*^*p* < 0.05, ^*##*^*p* < 0.01). *n* = 13, 10, 11 mice in PBS, LPS + siCTR, and LPS + siTPM1-1 groups, respectively

Triggering receptor expressed on myeloid cells 2 (TREM2), which is exclusively expressed by microglia, regulates neuroinflammation in the brain [20–23]. Interestingly, we observed that TREM2 deficiency upregulated TPM1 (Additional file 4: Fig. S4A, B), and TPM1 knockdown did not alter TREM2 expression level in LPS-treated in WT mouse retina (Additional file 4: Fig. S4C, D), suggesting that TPM1 might mediate inflammation downstream of TREM2. To further examine the relationship between TPM1 and TREM2 in inflammation process, we used TREM2 knockout mice. We found that LPS treatment remarkably increased the transcriptomic levels of TPM1, TNF-α, IL-1β, IL-6, COX-2 and iNOS in TREM2^−/−^ mouse retina compared to PBS-treated TREM2^−/−^ controls (Additional file 4: Fig. S4E, Fig. 3A–E). Surprisingly, we found that TPM1 knockdown further increased the expression levels of TNF-α, IL-1β, IL-6, COX-2 and iNOS (Additional file 4: Fig. S4E, Fig. 3A–E), and enhanced microglia and astrocyte activation in LPS-treated TREM2^−/−^ mouse retinas relative to siCTR- and LPS-treated TREM2^−/−^ controls (Fig. 3F–J). We found that TPM1 knockdown increased the numbers of activated microglia (Iba-1^+^CD68^+^) in the OPL of LPS-treated TREM2^−/−^ mouse retinas relative to siCTR- and LPS-treated group (Fig. 3F–H). Similarly, we observed that TPM1 knockdown promoted dendritic migration of more activated microglia into the ONL from the OPL in LPS-treated TREM2^−/−^ mouse retinas compared to LPS-treated TREM2^−/−^ control groups (Fig. 3I, Additional file 4: Fig. S4F). Moreover, we observed that LPS treatment increased GFAP immunoreactivity and the incidences of dendritic processes of activated astrocytes into the IPL from their normal NFL strata in TREM2^−/−^ mouse retinas compared to PBS-treated TREM2^−/−^ control mouse retinas (Fig. 3J, Additional file 4: Fig. S4G). Remarkably, we found that TPM1 knockdown further elevated GFAP immunoreactivity and astrocyte activation in LPS-treated TREM2^−/−^ mice compared to LPS-treated TREM2^−/−^ control mice (Fig. 3J, Additional file 4: Fig. S4G). Functionally, we observed decreases in the amplitudes of a- and b-waves of ERG in LPS-treated TREM2^−/−^ mice under both scotopic and photopic conditions (Fig. 3K, L). However, TPM1 knockdown further reduced a- and b-wave amplitudes of ERG in LPS-treated TREM2^−/−^ mice (Fig. 3K, L). Also, we found that TPM1 knockdown increased latency for a-waves under scotopic condition at 1 and 3 cd s/m^2^ light intensities in LPS-treated TREM2^−/−^ mice compared to PBS-treated TREM2^−/−^ mice (Additional file 4: Fig. S4H, I). Taken together, these results indicate that TPM1 knockdown aggravates LPS-induced inflammation and function decline in the TREM2^−/−^ mouse retina.

### TPM1 knockdown changes glial cell-specific transcriptomes in the TREM2^−/−^ mouse retina

**Fig. 4:**
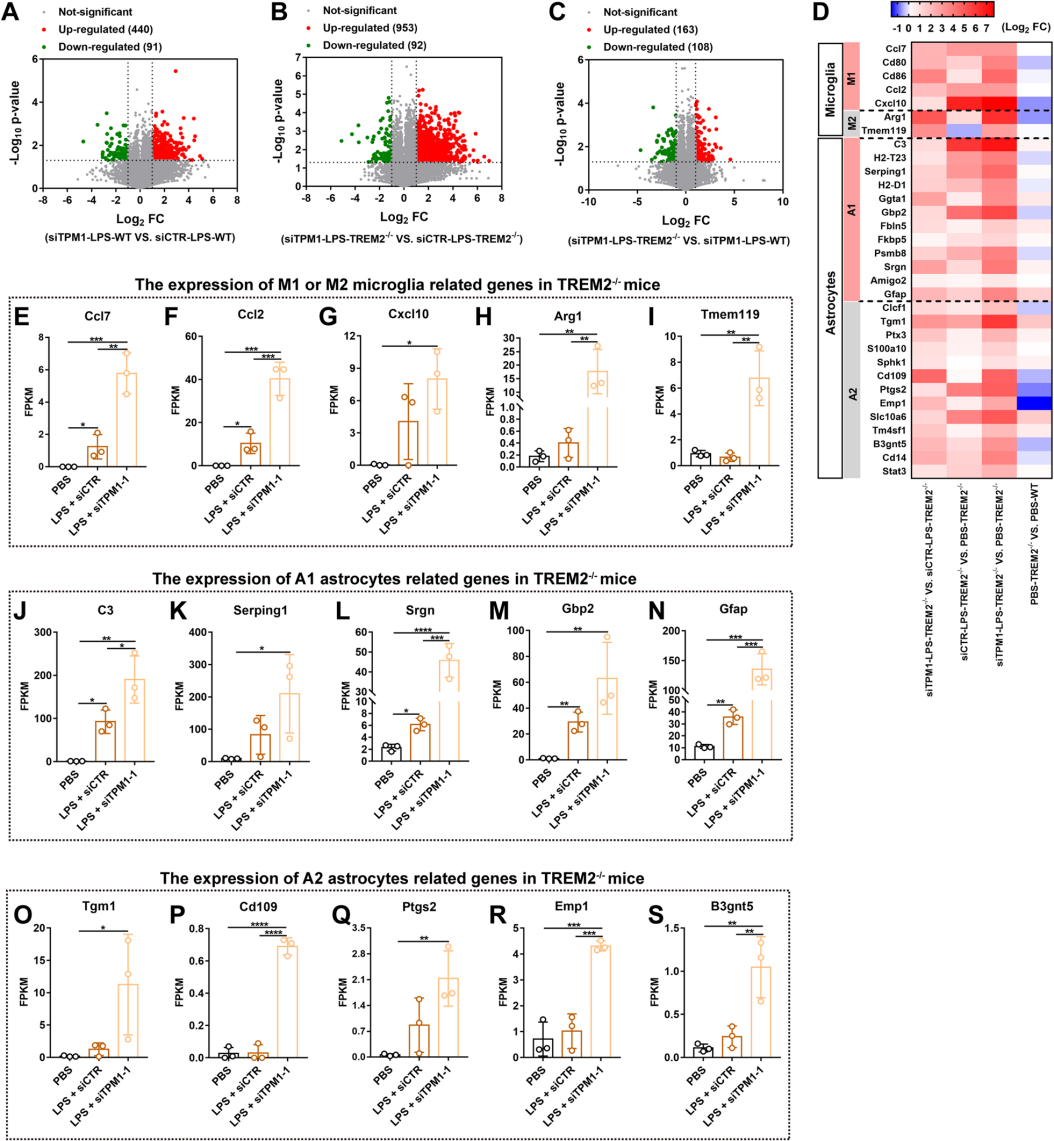
TPM1 knockdown changes transcriptome associated with glial cells in the TREM2^−/−^ mouse retina. **A**–**C** Volcano plots showing differentially expressed genes (DEGs) in WT or TREM2^−/−^ mice after treatments with LPS and siTPM1-1 or siCTR. Red dots indicate up-regulated genes and green dots show down-regulated genes (*n* = 3 mice in each group). **D** A heatmap showing DEGs associated with microglia and astrocytes in TREM2^−/−^ mice following treatments with PBS, or with LPS and siTPM1-1 or siCTR. **E**–**S** The expression of DEGs associated with M1 (**E**–**G**) or M2 microglia (**H**, **I**) and with A1 (**J**–**N**) or A2 astrocytes (**O**–**S**) in TREM2^−/−^ mice after treatments with LPS and siTPM1-1 or siCTR. Data are presented as mean ± SEM and analyzed by one-way ANOVA with Tukey’s multiple comparison test (compared to PBS or LPS + siCTR, *p < 0.05, **p < 0.01, ***p < 0.001, ****p < 0.001). *n* = 3 mice in each group. *FPKM* fragments per kilobase of transcript per million fragments mapped

We showed that TPM1 knockdown suppressed LPS-induced inflammation in the WT mouse retina but aggravated LPS-induced inflammation in the TREM2^−/−^ mouse retina. To decipher the differential roles of TPM1, we performed RNA sequences and subsequent transcriptome-wide analysis of genes and pathways in the TREM2^−/−^ or WT mouse retina with TPM1 knockdown. We found that 440 genes were upregulated and 91 genes were downregulated in siTPM1- and LPS-treated WT retinas compared to siCTR- and LPS-treated WT retinas (Fig. 4A). Meanwhile, 953 upregulated genes and 92 downregulated genes were identified in siTPM1- and LPS-treated TREM2^−/−^ mouse retinas compared to siCTR- and LPS-treated TREM2^−/−^ mouse retinas (Fig. 4B), suggesting that TPM1 knockdown induces robust gene expression changes in LPS-treated TREM2^−/−^ retinas. Indeed, we found that TPM1 knockdown promoted upregulation of more genes in LPS-treated TREM2^−/−^ retinas than in LPS-treated WT retinas (Fig. 4C). By analyzing differentially expressed genes (DEGs), we found that the genes associated with microglia and astrocytes were remarkably upregulated in LPS-treated WT retinas (Additional file 5: Fig. S5A, B, and E). However, we found that TPM1 knockdown decreased expression of the genes that were associated with M1 microglia (Cxcl10) and A1 astrocytes (Gbp2) (Additional file 5: Fig. S5A, B, and E), but increased expression of the genes related to M2 microglia (Arg1 and Temem119) and A2 astrocytes (Cd109 and Cd14) in LPS-treated WT retinas compared to siCTR- and LPS-treated WT control retinas (Additional file 5: Fig. S5A, C, D, F, and G), suggesting that TPM1 knockdown suppressed LPS-induced glial reactivity in WT retinas, which is consistent with our morphological observation (Fig. 2F–H). Similarly, we found that LPS treatment elevated the expression of DEGs which were associated with both microglia (M1 and M2) and astrocytes (A1 and A2) in TREM2^−/−^ mice (Fig. 4D–F, J, and L–N). Interestingly, we found that TPM1 knockdown further increased expression of the genes that were related to M1—(Ccl7, Ccl2 and Cxcl10) and M2—(Arg1 and Tmem119) microglia, and A1—(C3, Serping1, Srgn, Gbp2, Gfap) and A2—(Tgm1, Cd109, Ptgs2, Emp1 and B3gnt5) astrocytes in LPS-treated TREM2^−/−^ retinas compared to either siCTR- and LPS-treated TREM2^−/−^ control retinas or PBS-treated TREM2^−/−^ control retinas (Fig. 4D–S), suggesting that TPM1 knockdown further enhances glial cell reactivity in LPS-treated TREM2^−/−^ retinas, which is consistent with morphological alterations in microglia and astrocytes (Fig. 3F–J). Together, these results indicate that TPM1 knockdown increasingly induces transcriptomic alterations in microglia and astrocytes in LPS-treated TREM2^−/−^ retinas.

### TPM1-induced inflammation is microglia-dependent

**Fig. 5:**
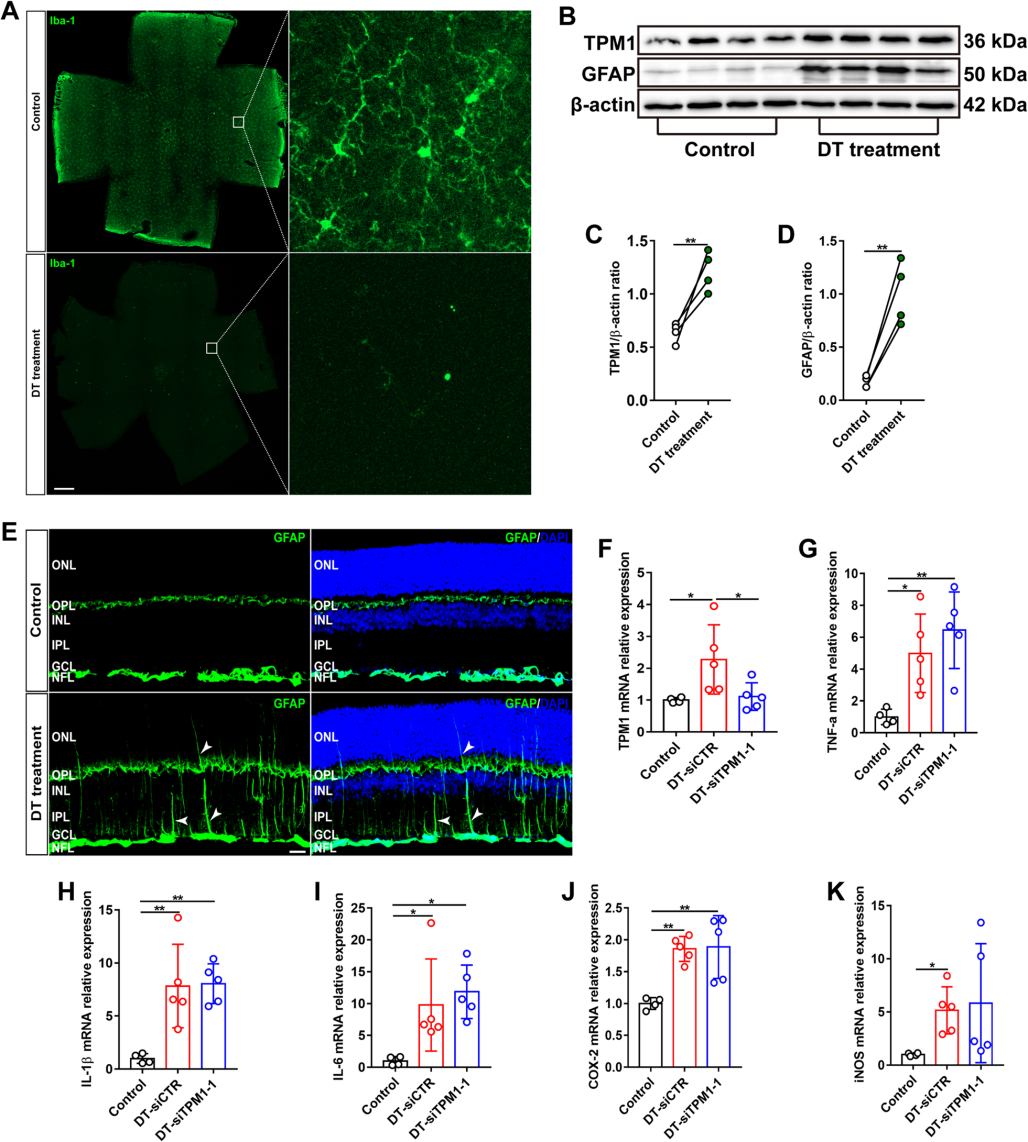
TPM1-induced inflammation is microglia-dependent. **A** Retinal whole-mounts from CX3CR1^CreER^:Rosa26^iDTR^ mice following tamoxifen (TAM) and subsequent diphtheria toxin (DT) treatments were stained with an antibody against Iba-1. Scale bar, 500 µm. **B**–**D** Western blot analysis (**B**) and quantification of TPM1 and GFAP (**C**, **D**) in microglia-depleted CX3CR1^CreER^:Rosa26^iDTR^ mouse retinas following TAM and DT treatment. Data are presented as mean ± SEM and analyzed by unpaired two-tailed Student’s *t* test (control vs. DT treatment, **p < 0.01). *n* = 4 mice in each group. **E** Retina sections stained with an antibody against GFAP in microglia-depleted CX3CR1^CreER^:Rosa26^iDTR^ mice following TAM and DT treatment. Arrowheads show activated astrocytes and Müller cells. Scale bars, 20 µm. **F**–**K** qPCR analysis of TPM1, TNF-α, IL-1β, IL-6, COX-2 and iNOS in microglia-depleted CX3CR1^CreER^:Rosa26^iDTR^ mice after siCTR or siTPM1-1 treatment. Data are presented as mean ± SEM and analyzed one-way ANOVA with Tukey’s multiple comparison test (compared to Control or DT-siCTR, *p < 0.05, **p < 0.01). *n* = 4, 5,5 mice in Control, DT-siCTR and DT-siTPM1-1 groups, respectively

To specifically investigate the role of microglia in TPM1-mediated inflammation, we genetically eliminated microglia from the retina. To this end, we crossed CX3CR1^CreER^ transgenic mice expressing tamoxifen-inducible Cre recombinase under the control of the CX3C chemokine receptor 1 promoter with Rosa26^iDTR^ mice expressing Cre-inducible diphtheria toxin receptor (iDTR), and generated a new CX3CR1^CreER^:Rosa26^iDTR^ mouse line. We conditionally depleted microglia from CX3CR1^CreER^:Rosa26^iDTR^ mice by administration of tamoxifen (TAM) and subsequent administration of DT 28–30 days later (Fig. 5A). We found that TPM1 was significantly elevated in the microglia-depleted retina of CX3CR1^CreER^:Rosa26^iDTR^ mice compared to CX3CR1^CreER^:Rosa26^iDTR^ control mice (Fig. 5B, C, and F), suggesting that microglia depletion triggers TPM1 upregulation. Moreover, we found that microglia depletion increased GFAP immunoreactivity (Fig. 5E), which was confirmed by WB analysis (Fig. 5B, D). Some processes of activated astrocytes migrated into the INL, even onto the ONL in microglia-depleted CX3CR1^CreER^:Rosa26^iDTR^ retinas (Fig. 5E). Meanwhile, we found that microglia deletion induced the upregulation of TNF-α, IL-1β, IL-6, COX-2 and iNOS (Fig. 5G–K). Consistently, previous studies reported that microglia depletion elevated inflammatory signatures and astrocytes activation [24–26]. Together these results indicate that microglia deletion induces TPM1 upregulation and inflammation. Surprisingly, we observed that TPM1 knockdown did not reduce the expression levels of these pro-inflammatory cytokines elicited by microglia deletion in CX3CR1^CreER^:Rosa26^iDTR^ retinas (Fig. 5F–K), suggesting that TPM1 regulates inflammation in a microglia-dependent manner.

### TPM1 knockdown elicits inflammation-related transcriptomic alterations and cell apoptosis in the TREM2^−/−^ mouse retina

**Fig. 6:**
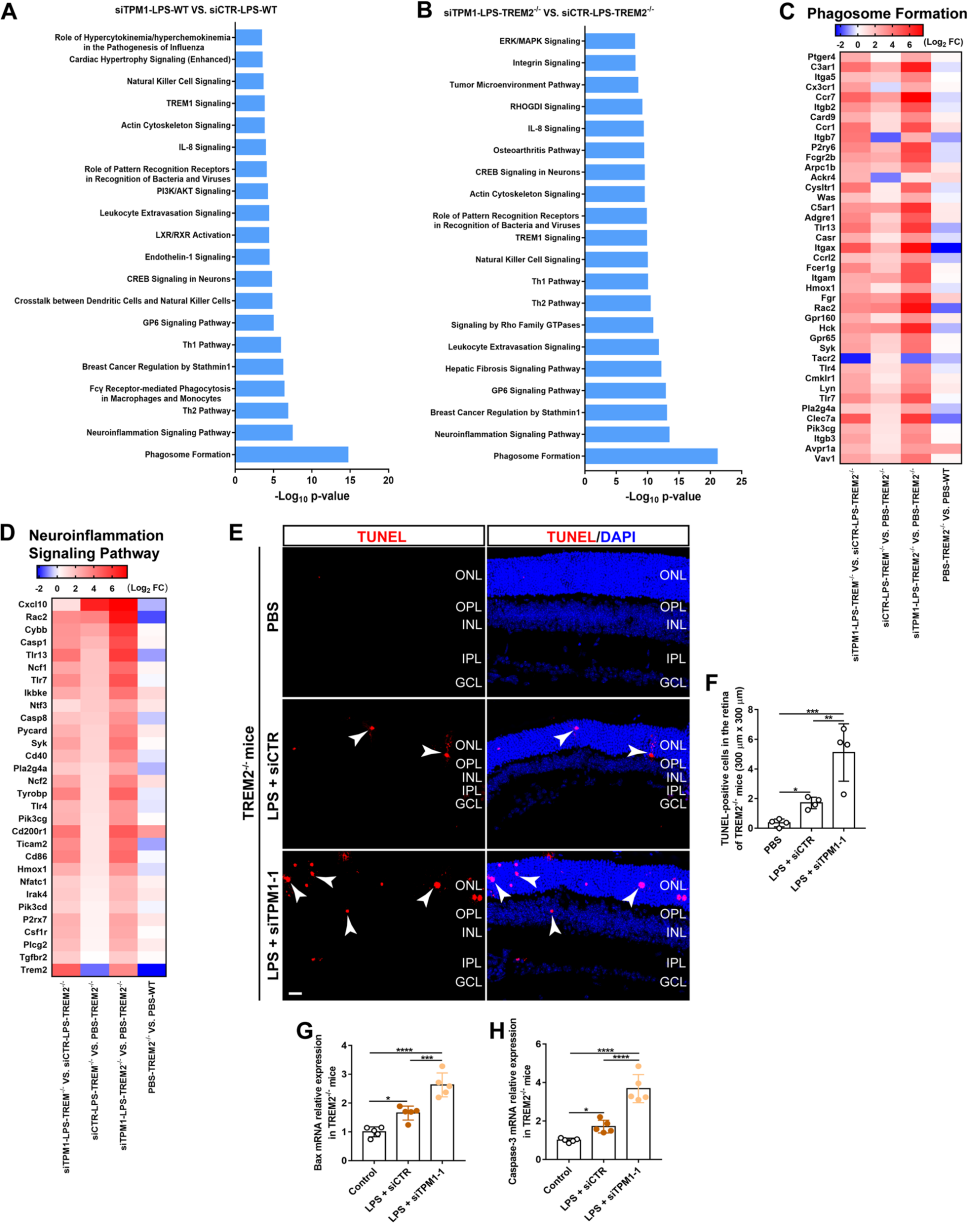
TPM1 knockdown elicits inflammation-related transcriptomic alterations and cell death in the TREM2^−/−^ retina. **A**, **B** Ingenuity Pathway Analysis (IPA) of top 20 canonical pathways in WT (**A**) or TREM2^−/−^ mice (**B**) after treatments with LPS and siTPM1-1 or siCTR. p < 0.001. **C**, **D** DEGs in the phagosome formation pathway (**C**) and neuroinflammation signaling pathway (**D**) in TREM2^−/−^ mice after treatments with LPS and siTPM1-1 or siCTR. **E**, **F**, TUNEL staining (**E**) and quantification of TUNEL-positive cells (**F**) in TREM2^−/−^ mice following treatments with PBS, or with LPS and siTPM1-1 or siCTR. Data are presented as mean ± SEM and analyzed by one-way ANOVA with Tukey’s multiple comparison test (compared to PBS or LPS + siCTR, *p < 0.05, **p < 0.01, ***p < 0.001). *n* = 4 mice in each group. **G**, **H** qPCR analysis of Bax and Caspase-3 in TREM2^−/−^ mouse retinas after treatments with LPS and siTPM1-1 or siCTR. Data are presented as mean ± SEM and analyzed by one-way ANOVA with Tukey’s multiple comparison test (compared to PBS or LPS + siCTR, *p < 0.05, ***p < 0.001, ****p < 0.001). *n* = 5 mice in each group

Ingenuity pathway analysis of DEGs revealed that most of DEGs were strongly associated with inflammation process, including phagosome formation, neuroinflammation signaling pathway in pairwise comparisons between LPS-treated WT with or without TPM1 knockdown (Fig. 6A), and between LPS-treated TREM2^−/−^ mice with or without TPM1 knockdown (Fig. 6B). Among these DEGs, we found that Myl1, Oprk1, Myh6, Myo10, Ptgdr2, which are related to phagocytosis, were downregulated in siTPM1- and LPS-treated WT retina but upregulated in siCTR- and LPS-treated WT control retinas (Additional file 6: Fig. S6A), suggesting that TPM1 knockdown boosts phagocytic activity suppressed by LPS in WT mice. Interestingly, we found that LPS treatment triggered overexpression of phagosome formation related genes in TREM2^−/−^ mice (Fig. 6C), including C3ar1, Ccr7, Itgb2, Ccr1, P2ry6, Itgax, Fgr, Rac2, Hck, Clec7a, and those genes were further elevated in siTPM1- and LPS-treated TREM2^−/−^ mice, suggesting that TPM1 knockdown results in the enhancement of LPS-induced autophagy in TREM2^−/−^ mice. Among induced DEGs that were related to the neuroinflammation signaling pathway, we found that LPS significantly elevated expressions of pro-inflammatory cytokines including Cxcl10, CD40 and CD86 in WT mice compared to WT control mice (Additional file 6: Fig. S6B). However, we found that TPM1 knockdown remarkably inhibited Cxcl10 (Additional file 6: Fig. S6B) and improved anti-inflammatory cytokines including Csf1r, Irak4 and Trem2 in LPS-treated WT compared to siCTR- and LPS-treated WT control mice (Additional file 6: Fig. S6B), suggesting that TPM1 knockdown inhibits LPS-induced neuroinflammation in WT mice, which is consistent with our in vitro (Additional file 2: Fig. S2A–G) and in vivo (Fig. 2A–H) results. Furthermore, we found that LPS significantly increased inflammatory mediators including Cxcl10, Rac2, Cybb, Casp1, Tlr13, Ncf1 and Tlr7 in TREM2^−/−^ mice (Fig. 6D), and those genes were further elevated in LPS-treated TREM2^−/−^ mice following siTPM1 treatment, indicating that TPM1 knockdown leads to the enhancement of LPS-induced neuroinflammation in TREM2^−/−^ mice, which is consistent with our in vivo results (Fig. 3A–J).

To examine whether the increased inflammation induced by TPM1 knockdown resulted in more cell apoptosis in LPS-treated TREM2^−/−^ mice, we performed TUNEL staining. We observed that LPS significantly increased the numbers of TUNEL-positive cells in siCTR-treated TREM2^−/−^ retinas (3.73-fold) compared to PBS-treated TREM2^−/−^ control retinas (Fig. 6E, F). Interestingly, we found that TPM1 knockdown caused more TUNEL-positive cells (1.98-fold) in LPS-treated TREM2^−/−^ retinas than in siCTR- and LPS-treated TREM2^−/−^ control retinas (Fig. 6E, F), suggesting that TPM1 knockdown aggravates cell apoptosis in LPS-treated TREM2^−/−^ mice. Mechanistically, we found that LPS treatment significantly increased mRNA levels of Bax (65.2%) and Caspase-3 (70.66%), which are associated with cell apoptosis, in siCTR-treated TREM2^−/−^ retinas compared to PBS-treated TREM2^−/−^ control retinas (Fig. 6G, H). TPM1 knockdown further increased expression of Bax (59.2%) and Caspase-3 (115.9%) in LPS-treated TREM2^−/−^ retinas compared to siCTR- and LPS-treated TREM2^−/−^ mouse retinas (Fig. 6G, H).

Collectively, these results suggest that TPM1 knockdown promotes additional increase in neuroinflammation and more cell apoptosis in TREM2-deficient mouse retinas following LPS treatment.

### TPM1 knockdown dysregulates the PKA/CREB signaling pathway in the TREM2^−/−^ mouse retina

**Fig. 7:**
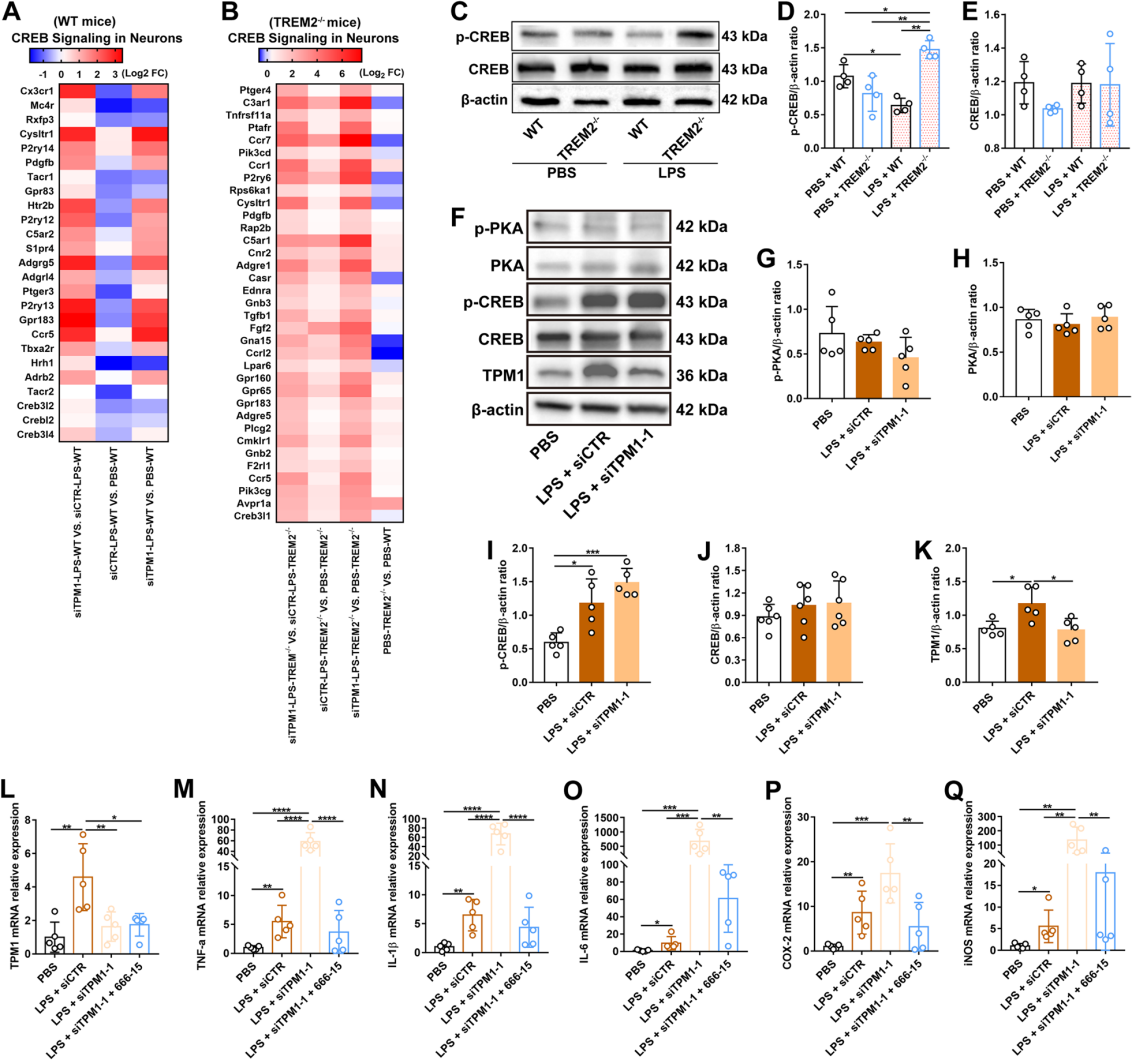
TPM1 knockdown dysregulates the PKA/CREB pathway in the TREM2^−/−^ retina. **A**, **B** DEGs related to the CREB signaling in neurons in WT (**A**) or TREM2^−/−^ mice (**B**) following treatments with PBS, or with LPS and siTPM1-1 or siCTR. **C**–**E** Western bot analysis (**C**) and quantification of p-CREB and CREB (**D**, **E**) in WT or TREM2^−/−^ mice following LPS or PBS treatment. Data are presented as mean ± SEM and analyzed by one-way ANOVA with Tukey’s multiple comparison test (compared to LPS + TREM2^−/−^, *p < 0.05, **p < 0.01; PBS + WT vs. LPS + WT, *p < 0.05). *n* = 4 mice in each group. **F**–**K** Western blot analysis (**F**) and quantification of p-PKA, PKA, p-CREB, CREB and TPM1 (**G**–**K**) in TREM2^−/−^ mice after treatments with LPS and siTPM1-1 or siCTR. Data are presented as mean ± SEM and analyzed by one-way ANOVA with Tukey’s multiple comparison test (compared to PBS or LPS + siCTR, *p < 0.05, ***p < 0.001). *n* = 5 or 6 mice in each group. **L**–**Q** qPCR analysis of TPM1, TNF-α, IL-1β, IL-6, COX-2 and iNOS in TREM2^−/−^ mice after treatments with LPS, siTPM1-1 and 666-15, a potent and selective CREB inhibitor. Data are presented as mean ± SEM and analyzed by one-way ANOVA with Tukey’s multiple comparison test (compared to LPS + siCTR or LPS + siTPM1-1, *p < 0.05, **p < 0.01, ***p < 0.001, ****p < 0.0001). *n* = 5 mice in each group

Ingenuity pathway analysis of DEGs revealed that CREB signaling in neurons was involved in LPS-treated WT or TREM2^−/−^ mouse retinas following TPM1 knockdown (Fig. 7A, B). Among induced genes associated with the CREB signaling pathway, Cx3cr1, Mc4r, Ptger3, Hrh1, Tacr2, Creb3l2 and Creb3l4, were downregulated in siCTR- and LPS-treated WT retinas but upregulated in siTPM1- and LPS-treated WT retinas (Fig. 7A), suggesting that TPM1 knockdown reverses LPS-induced inhibition of the CREB signaling pathway. Indeed, our in vitro (Additional file 2: Fig. S2H–Q) and in vivo (Fig. 2K–P) results also verified that TPM1 knockdown counteracted the decline of p-CREB induced by LPS treatment. Interestingly, we found that the vast majority of genes that are associated with the CREB signaling pathway, such as C3ar1, Ccr1, Ccr7, P2ry6, C5ar1, Fgf2 and Gpr65, were overexpressed in LPS-treated TREM2^−/−^ mouse retinas relative to PBS-treated TREM2^−/−^ control retinas (Fig. 7B), suggesting that LPS activates the CREB signaling pathway in TREM2^−/−^ mice. Evidently, western blot analysis revealed elevated production of p-CREB in LPS-treated TREM2^−/−^ retinas (Fig. 7C–E). TPM1 knockdown further increased the expression level of the genes associated with the CREB signaling pathway in LPS-treated TREM2^−/−^ retinas compared to siCTR- and LPS-treated TREM2^−/−^ mouse retinas (Fig. 7B), suggesting that TPM1 knockdown further enhances the activation of the CREB signaling pathway induced by LPS in TREM2^−/−^ mice. Furthermore, we observed that LPS administration significantly elevated the expression of p-CREB but not p-PKA and PKA in TREM2^−/−^ retinas (Fig. 7F–J), indicating the dysregulation of the PKA/CREB signaling pathway in LPS-treated TREM2^−/−^ mice. After TPM1 knockdown, p-CREB also showed an increase trend in LPS-treated TREM2^−/−^ retinas relative to siCTR- and LPS-treated TREM2^−/−^ control retinas (Fig. 7F–K), even though p-CREB expression level was not statistically significant between two groups, suggesting that TPM1 knockdown dysregulates the PKA/CREB signaling pathway in LPS-treated TREM2^−/−^ retinas.

To confirm the specific function of CREB in TREM2^−/−^ retinas, we treated TREM2^−/−^ mice with LPS, siTPM1 and 666-15, a potent and selective CREB inhibitor. After CREB inhibition (Additional file 7: Fig. S7A–C), we found that TPM1 knockdown failed to increase pro-inflammatory cytokines (TNF-α, IL-1β and IL-6) and chemokines (COX-2 and iNOS) in LPS-treated TREM2^−/−^ mice compared with siCTR- and LPS-treated TREM2^−/−^ control mice (Fig. 7L–Q). These results confirm that CREB promotes pro-inflammatory responses in the TREM2^−/−^ retina through overexpression of pro-inflammatory cytokines, such as IL-6, which has been associated with inflammatory and neurodegenerative processes [27, 28]. Our findings demonstrate that CREB regulate pro-inflammatory response in the TREM2^−/−^ retinas.

Collectively, these results indicate that TPM1 knockdown deteriorates neuroinflammation in TREM2-deficient mouse retinas following LPS treatment by dysregulating the PKA/CREB signaling pathway.

### TPM1 mediates inflammatory responses downstream of TREM2

**Fig. 8:**
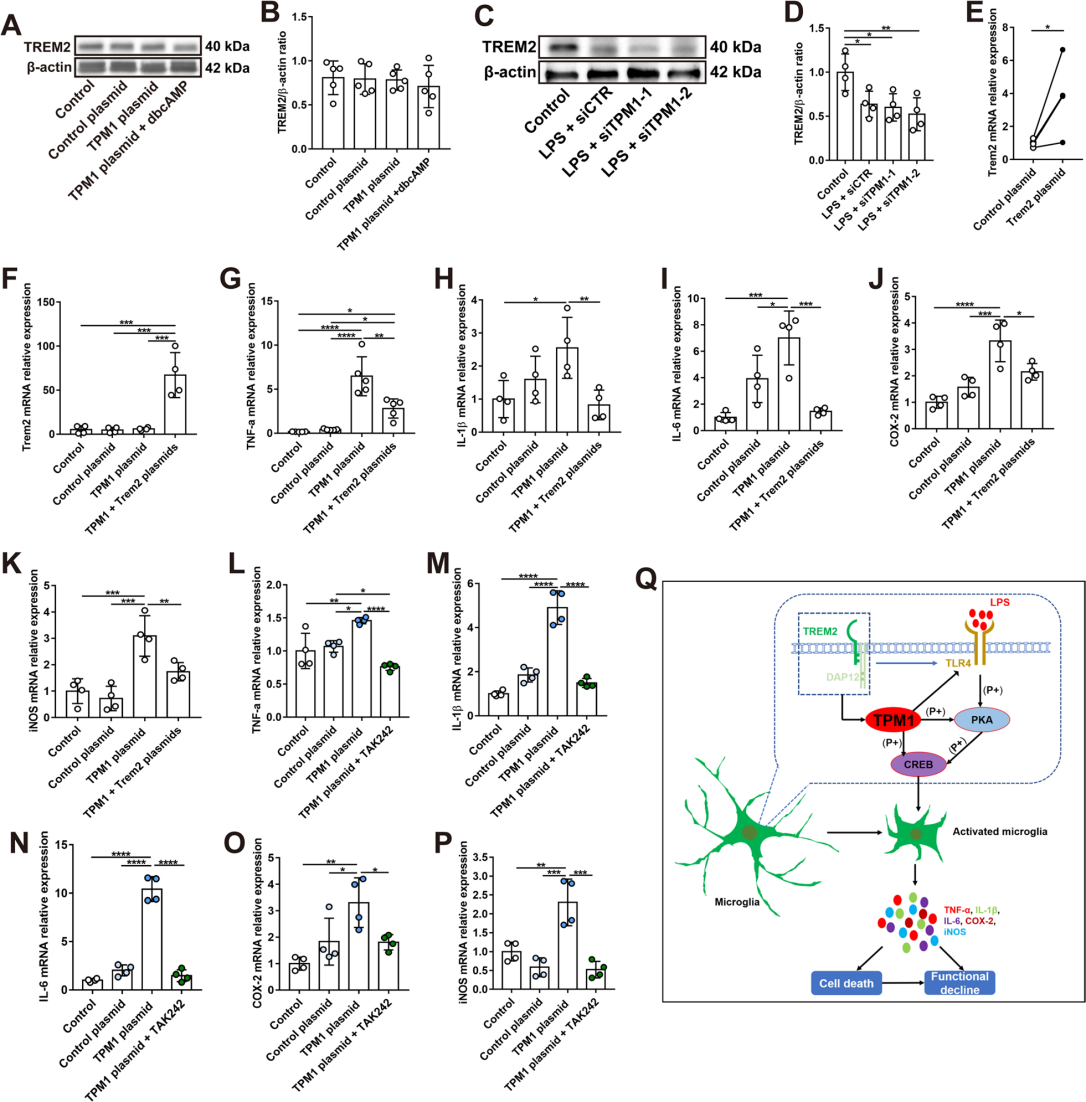
TPM1 mediates inflammatory responses downstream of TREM2. **A**, **B** Western blot analysis (**A**) and quantification of TREM2 (**B**) in BV2 cells after treatments with control plasmid, TPM1 plasmid, or TPM1 plasmid and dbcAMP, a PKA activator. Data are presented as mean ± SEM. **C**, **D** Western blot analysis (**C**) and quantification of TREM2 (**D**) in BV2 cells following treatments with LPS and siTPM1-1, siTPM1-2 or siCTR. Data are presented as mean ± SEM and analyzed by one-way ANOVA with Tukey’s multiple comparison test (compared to Control, *p < 0.05, **p < 0.01). Four independent experiments were performed. **E** mRNA level of Trem2 in BV2 cells after transfection with Trem2 plasmid. Data are presented as mean ± SEM, unpaired two-tailed Student’s *t* test (control plasmid vs. Trem2 plasmid, *p < 0.05). **F**–**K** qPCR analysis of Trem2, TNF-α, IL-1β, IL-6, COX-2 and iNOS in BV2 cells following transfection with control plasmid, TPM1 plasmid, or TPM1 and Trem2 plasmids. Data are presented as mean ± SEM and analyzed by one-way ANOVA with Tukey’s multiple comparison test (compared to TPM1 plasmid or TPM1 + Trem2 plasmids, *p < 0.05, **p < 0.01, ***p < 0.001, ****p < 0.001). Four independent experiments were performed. **L**–**P** qPCR analysis of TNF-α, IL-1β, IL-6, COX-2, iNOS and Trem2 in BV2 cells after treatments with control plasmid, TPM1 plasmid, or TPM1 plasmid and TAK242, a specific inhibitor of TLR4. Data are presented as mean ± SEM and analyzed by one-way ANOVA with Tukey’s multiple comparison test (compared to TPM1 plasmid or TPM1 plasmid + TAK242, *p < 0.05, **p < 0.01, ***p < 0.001, ****p < 0.001). Four independent experiments were performed. **Q** Schematic depicting TPM1-regulated pathways in neuroinflammation. TPM1 signals downstream of TREM2 and regulates neuroinflammation and neuronal cell death by mediating the CREB signaling pathways and in a manner of microglia-dependent

We observed that TREM2 affected the role of TPM1 in regulating inflammation. Therefore, we examined whether TPM1 acted downstream of TREM2. We initially transfected BV2 cells with TPM1 plasmid and found that TPM1 overexpression did not affect the expression of TREM2 (Fig. 8A, B), suggesting that TREM2 might be an upstream molecule of TPM1. Moreover, we observed that LPS treatment decreased the protein level of TREM2 in BV2 cell (Fig. 8C, D), which is in line with previous observations [29, 30], and TPM1 knockdown did not reverse this trend (Fig. 8C, D), further suggesting that TREM2 signals upstream of TPM1. To confirm this possibility, we co-transfected BV2 cells with both TREM2 and TPM1 plasmids (Fig. 8E, F; Additional file 8: Fig. S8A–D). We observed that TPM1 overexpression increased the expression levels of pro-inflammatory cytokines (Fig. 8G–K), which were downregulated by TREM2 overexpression (Fig. 8G–K), indicating that TREM2 potentially regulates TPM1-induced inflammation. Indeed, we found that co-transfection of TPM1 and TREM2 plasmids significantly reduced TPM1 expression relative to transfection of TPM1 alone (Additional file 8: Fig. S8A–D), suggesting that TPM1 might regulate inflammatory responses downstream of TREM2.

TREM2 is previously reported to modulate Toll-like receptor 4 (TLR4)-induced inflammation [11, 13, 21, 31]. Interestingly, our transcriptomic analysis also revealed that TPM1 knockdown significantly increased TLR4 expression in LPS-treated TREM2^−/−^ mice (Fig. 6D), suggesting that TLR4 could be involved in TPM1-mediated inflammation in TREM2^−/−^ mice. To further confirm the interaction between TPM1 and TLR4, we transfected BV2 cells with TPM1 plasmid and then treated the cells with TAK242, a specific inhibitor of TLR4. We found that TPM1 overexpression failed to increase expression levels of pro-inflammatory cytokines and chemokines (Fig. 8L–P), suggesting the direct involvement of TLR4 in TPM1-induced neuroinflammation. Collectively, these results indicate that TPM1 might regulate inflammation downstream of TREM2 and via TLR4.

Taken together, our data demonstrated that TPM1 was involved in LPS-induced inflammation and functional decline via mediating the PKA/CREB signaling pathway (Fig. 8Q). Moreover, TPM1 regulated LPS-induced inflammation and neuronal cell death downstream of the TREM2 signal and in a microglia-dependent manner (Fig. 8Q). These results suggest that TPM1 could be a potential target to combat inflammation in the diseased retina.

## Discussion

In this study, we demonstrated in addition to its crucial roles in the regulation of neurite outgrowth, branching and synapse formation in neurons [32], the actin-associated protein TPM1 played a previously unanticipated role in regulating pro-inflammatory genes in microglia. We demonstrated that TPM1 overexpression triggered microglia activation and the release of pro-inflammatory cytokines, and TPM1 knockdown ameliorated LPS-induced inflammation in our in vitro studies. Our in vivo studies confirmed in vitro results and showed that TPM1 was involved in LPS-activated inflammation in microglia and facilitated LPS-induced inflammation and function decline in the WT retina. We further showed that TPM1 exerted its effect via the PKA/CREB pathway in the WT retina. Previous studies have reported that CREB acts as a transcription factor that regulates the expression of genes involved in immune function, including inhibition of inflammation [16, 17], which is consistent with our observation.

As an important innate immune receptor in the brain, TREM2 has previously been shown to promote proliferation, phagocytosis, and migration of microglia by induction and maintenance of microglial activation [12, 29, 33]. Loss of TREM2 leads to reduced phagocytosis of apoptotic cells and causes aberrant neuroinflammation [21, 34]. This effect is consistent with our results in which TREM2 knockout resulted in upregulation of inflammatory mediators in response to LPS stimulation. Interestingly, we observed that TPM1 was upregulated in TREM2^−/−^ mouse retina, and that TREM2 regulated TPM1 and TPM1-induced inflammation. Surprisingly, we found that TPM1 knockdown did not reduce but exaggerated LPS-induced inflammation in the TREM2^−/−^ mouse retina. Moreover, we observed that TPM1 knockdown induced additional production of inflammatory mediators and caused extra neuronal cell death and function decline in LPS-treated TREM2^−/−^ mice when compared with siCTR- and LPS-treated TREM2^−/−^ control mice. Collectively, we demonstrate that TPM1 functions downstream of TREM2, and that TPM1 knockdown promotes an extra boost to LPS-mediated inflammation and neuronal cell death in the absence of TREM2.

To decipher the differential roles of TPM1 in the retina with or without TREM2, we performed transcriptome-wide analysis of genes and revealed that TPM1 knockdown decreased expression of the genes associated with detrimental M1 microglia and A1 astrocytes in LPS-treated WT retinas. Our results are in agreement with previous reports that showed that M1 microglia and A1 astrocytes modulate pro-inflammatory and neurotoxic activities in the CNS [35–38]. Reactive glial cells including microglia and astrocytes are reported to induce neuronal death in neurological disorders [35–38]. For instance, A1 reactive astrocytes are demonstrated to be involved in neurodegeneration and disease progression in a variety of human diseases, including AD [37–39]. Similarly, LPS stimulation elevated the expression of differentially expressed genes (DEGs) associated with M1 microglia and A1 astrocytes in the TREM2^−/−^ mouse retina compared to TREM2^−/−^ control retinas. Interestingly, the upregulation of the DEGs was further amplified by TPM1 knockdown in LPS-treated TREM2^−/−^ retinas, eventually leading to enhanced reactivity of microglia and astrocytes, the exaggerated production of inflammatory mediators and additional neuronal cell death and function decline in TREM2^−/−^ mice, when compared to siCTR- and LPS-treated TREM2^−/−^ control retinas. Previous studies reported that TREM2 depletion impaired microglia migration toward beta-amyloid deposits [40, 41]. Meanwhile, other studies showed that LPS treatment induced TREM2 downregulation and microglia migration [29, 42, 43]. Similarly, our results showed that TREM2 deficiency promoted microglia migration towards the ONL and RPE layers in the retina following LPS treatment (Fig. 3I), indicating that other mechanisms induced by LPS stimulation are responsible for microglia migration after TREM2 deficiency. Taken together, our data indicate that TPM1 induces neuronal cell loss in part by facilitating the production of neurotoxic mediators by microglia and astrocytes. Moreover, CD68 expression is reported to be associated with TREM2 in the brain [44]. However, we observed increased expression of CD68 in both WT and TREM2^−/−^ mice after LPS application, suggesting that CD68 expression might be TREM2-independent.

Moreover, we observed that LPS induced CREB activation in TREM2^−/−^ retinas. TPM1 knockdown further enhanced the activation of the CREB pathway in LPS-treated TREM2^−/−^ mouse retinas, and CREB then increased production of pro-inflammatory cytokines, such as IL-6. IL-6 is previously reported to be associated with inflammatory and neurodegenerative processes [27, 28, 45, 46]. After CREB inhibition, however, we found that TPM1 knockdown failed to induce expression of pro-inflammatory cytokines including IL-6 in LPS-treated TREM2^−/−^ retinas, confirming that IL-6 activated by CREB might contribute to TPM1-regulated inflammation in absence of TREM2. Meanwhile, we demonstrated that TPM1 knockdown resulted in the induction of anti-inflammatory cytokines, such as IL-10, via its ability to trigger activation of CREB [47, 48], which in turn inhibited LPS-induced inflammation and prevented neuronal cell loss in WT retinas. Collectively, we have revealed two distinct roles of CREB in mediating the induction of pro-inflammatory genes in TREM2^−/−^ retinas and anti-inflammatory genes in WT retinas. More importantly, we had revealed a new role for TREM2 as an important signaling molecule that potentially serves as a critical negative regulator of TPM1-mediated neuroinflammation. Consistently, CREB is previously reported to play many different roles in immune function, and CREB mediates pro-inflammatory genes, such as IL-6 [45, 46], and anti-inflammatory genes, such as IL-10 [47, 48], as well. Previous transcriptomic analysis revealed that TREM2 knockout induces cell death by regulating mTOR, MAPK and ERK signaling pathways, and mediates cell migration by modulating CXCR4 signal [33, 40]. It warrants further investigation to reveal whether these potential pathways are involved in regulating inflammation and cell death in the retina in the future.

Previous studies had reported that TREM2 modulates Toll-like receptor 4 (TLR4) and TLR4-driven inflammation [12, 29, 33], and that CREB plays a specific role in the LPS/TLR4 pathway [16, 17, 45, 47]. We observed upregulation of TLR4 and the CREB signaling pathway in LPS-treated TREM2^−/−^ mouse retinas, and identified that TPM1 regulated LPS-induced inflammation downstream of TREM2. Meanwhile, we observed that TPM1 overexpression increased expression levels of pro-inflammatory cytokines and chemokines but failed to induce the inflammation in the presence of TAK242, a specific inhibitor of TLR4, suggesting the direct involvement of TLR4 in TPM1-regulated inflammation. Collectively, TREM2 might mediate TPM1 signaling and neuroinflammation through regulating TLR4 receptors in the retina. Our findings revealed an important role for TPM1 in driving LPS-mediated inflammation, CREB activation and induction of CREB-responsive genes via TREM2 and TLR4.

The present study demonstrates a potent inflammatory activity of TPM1 in microglia and astrocytes. It appears that TPM1 alters gene signatures in both microglia and astrocytes, which shift them from a homeostatic state to a disease-associated state that promote inflammation and neuronal cell death. To confirm the possibility, we eliminated microglia from the retina. We found that microglia elimination dramatically increased pro-inflammatory responses in the retina, which is consistent with previous reports that microglia depletion increases inflammatory signatures and astrocytes activation in the brain [24–26]. In the absence of microglia, we observed TPM1 upregulation, which is probably contributed by retinal neurons and/or other type of glial cells, such as, reactive astrocytes. Consistently, previous studies reported TPM1 expression in neurons of the brain [49, 50]. However, we observed that TPM1 knockdown failed to suppress the inflammation elicited by microglia depletion, suggesting that the inflammation elicited by microglia depletion is mediated probably by other type of glial cells via other signaling pathways, such as astrocytes, and that TPM1 potentially regulates inflammation majorly through microglia. For instance, A1 reactive astrocytes are involved in disease progression in a variety of human brain diseases, including AD [37–39]. Also, we cannot eliminate the involvement of other cellular processes or signaling pathways in TPM1-regulated inflammation after microglia depletion. Additional experiments will be needed to test the possibility. Our data indicate that TPM1 is an important regulator of pro-inflammatory genes in microglia, which can trigger changes in gene signatures in microglia, resulting in a gradual transition from homeostatic microglia to disease-associated microglia.

More recently, we have reported TPM1 elevation in aged retinas and in the retinas of AD mouse models [7], indicating the potential pathological role of overexpressed TPM1 in neurodegenerative diseases. In this study, we demonstrated that TPM1 overexpression regulates neuroinflammation. Increasing evidences have shown that inflammation is involved in a different kind of retinal diseases including age-related macular degeneration, diabetic retinopathy, retinal vein occlusion, and retinitis pigmentosa [51, 52]. It will be interesting to investigate the role of TPM1 in regulating inflammation in these retinal diseases and AD in the future.

In conclusion, our findings reveal a vital role for TPM1 in facilitating LPS-mediated inflammation in microglia and surrounding neuronal cell death through the PKA/CREB pathway. Moreover, TPM1 exerts its effects downstream of the TREM2 signal and in a microglia-dependent manner. More importantly, we identify a novel role for TREM2 serving as a brake on TPM1-mediated inflammation. In absence of TREM2, TPM1 knockdown activates CREB, allowing for overexpression of CREB-mediated pro-inflammatory genes. Therefore, manipulation in TPM1 expression by either a pharmacological intervention targeting specific TPM1 or virus-mediated suppression of TPM1 could provide a unique avenue to gently restore balance in disrupted TPM1 systems to slow down the pathogenetic progression in neurodegenerative diseases including AD.

## Methods

### Mice

C57BL/6 mice (Stock no: 000664), TREM2^−/−^ mice (Stock no: 027197), CX3CR1^CreER^ mice (Stock no: 021160) and Rosa26^iDTR^ mice (Stock no: 007900) were obtained from the Jackson Laboratory, USA. CX3CR1^CreER^ mice were used to cross with Rosa26^iDTR^ mice to generate CX3CR1^CreER^:Rosa26^iDTR^ for conditionally ablating microglia. All mice were housed in a 12 h light/dark cycle with water and food ad libitum, and maintained in the Centralised Animal Facilities, The Hong Kong Polytechnic University. All experimental procedures were approved by the Animal Subjects Ethics Sub-committee (ASESC) of The Hong Kong Polytechnic University and conducted in accordance with the ARVO statement for the use of animals.

### Plasmid construction and siRNA synthesis

TPM1 and Trem2 plasmids and the corresponding control plasmids were constructed by UBIGENE (USA) and Synbio Technologies (China), respectively. In brief, the 846 bp fragments of TPM1 (NM_001164255.1) and the 698 bp fragments of TREM2 (NM_031254.3) from the start codon were subcloned into the vector p-YOE-PR006 (UBIGENE) and the vector p-CMV-myc, respectively, followed by restriction enzyme analysis and sequencing. Three siRNA pools targeting TPM1 (siTPM1-1) were synthesized by Synbio Technologies (China). The Silencer® Select siRNA targeting TPM1 (siTPM1-2) was purchased from Thermo Fisher Scientific (Hong Kong). Detailed siRNA sequences are listed in Additional file 10: Table S1.

### Plasmids and siRNA transfection

BV2 cells were seeded in six-well plate at a concentration of 5 × 10^5^ cells per well, and cultured with Dulbecco’s modified Eagle’s media (DMEM) containing 10% FBS, 1% penicillin/streptomycin and 2 mM l-Glutamine in a 37 °C incubator under 5% CO_2_ as described in our previous studies [32]. For plasmid transfection, 2 ug plasmid was co-transfected with 8 µl *TransIntro™* PL Transfection Reagent (TransGen, China) or Lipofectamine 3000 (Invitrogen) in serum-free Opti-MEM™ medium (Thermo Fisher, USA) for 4–6 h, followed by changing complete culture medium. After 2 days, cells were collected to validate transfection efficiency by qRT-PCR. For siRNA transfection, 10 µm siRNA was transfected with 9 µl Lipofectamine® RNAiMAX Reagent (Life Technologies) on BV2 cells, and knocking-down efficiency was confirmed at 2nd of post-transfection by qRT-PCR. For the signaling pathway study, cells were transfected by plasmids or siRNA for 24 h before treatment with LPS (1 µg/ml) and H89 (10 µM), dbcAMP (1 mM) and TAK242 (1 µM) for 1 day, and then cells were collected for further analysis.

### Intravitreal siRNA injections

The intravitreal injection of siRNA was performed as previously described [53–55]. After anesthesia with a mixture of ketamine hydrochloride (100 mg/kg) and xylazine (20 mg/kg), mouse was put under a dissecting microscope and one drop of 0.5% alcaine was applied to the eye. Under microscopic control, an incision was made into the superior nasal sclera using a sterile, sharp 31G needle. After removing the needle, a glass pipette was carefully inserted into the same incision, and around 1 µl siRNA solution was slowly injected into the vitreous. The pipette was kept inside the vitreous for a few seconds after the injection to prevent reflux of siRNA solution. After injections, antibiotic eye ointment was applied to prevent infection. 100 µM TPM1 siRNA or negative control siRNA was intravitreally injected to the retina of C57BL/6J or TREM2^−/−^ mice for three times, 3 days apart. The knockdown efficiency of TPM1 was evaluated by qPCR.

### LPS and 666-15 treatments in vivo

666-15 (MedChemExpress) was dissolved in 10% DMSO and 90% corn oil, and TREM2^−/−^ mice (4–5 weeks) were treated with a vehicle or 666-15 at 20 mg/kg via intragastric gavage for five times, 2 days apart. The inhibition efficiency of CREB signaling pathway by 666-15 treatment was evaluated by Western blot. After siRNA or 666-15 treatments, LPS (Sigma) at 5 mg/kg was intraperitoneally injected to C57BL/6J or TREM2^−/−^ mice, and retinas were collected for further analysis in the following day.

### Microglia depletion

Tamoxifen (TAM, Sigma) was dissolved in corn oil at 37 °C for 2 h. CX3CR1^CreER^:Rosa26^iDTR^ mice were treated with 10 mg of TAM for twice by gavage, 2 days apart. After 28–30 days, animals were intraperitoneally injected with Diphtheria toxin (DT, Sigma) at 10 µg/kg for 3 consecutive days to deplete microglia cells.

### Immunoblotting

After different treatments, cells or retinas were homogenized by RIPA buffer (Abcam) or T-PER™ Tissue Protein Extraction Reagent (Invitrogen) containing the cocktails of protease and phosphatase inhibitors (Roche), and the supernatant was transferred to a new tube for protein quantification with Pierce™ rapid gold BCA protein assay kit (Invitrogen) after centrifugation at 10,000×*g* for 15 min. Western blot analysis was performed as previously described [32, 56]. In brief, twenty-five microgram of protein was subjected to 10% SDS-PAGE gel, and then transferred to a polyvinylidene difluoride membrane. Following primary antibodies were used: rabbit anti-TPM1 (ABclonal, 1:1000), rabbit anti-Phospho-PKA C (Thr197) (Cell Signaling Technology, 1:1000), rabbit anti-PKA C-α (Cell Signaling Technology, 1:1000), rabbit Phospho-CREB (Ser133) (Cell Signaling Technology, 1:1000), rabbit CREB (48H2) (Cell Signaling Technology, 1:1000), CD68 (Bio-rad, 1:500), or mouse anti-actin (ABclonal, 1:2000). Goat anti-rabbit IgG, goat anti-rat IgG and goat anti-mouse IgG (Invitrogen, 1:1000) conjugated to horseradish peroxidase were applied as secondary antibodies.

### Immunocytochemistry and confocal imaging

After enucleation, the retina was separated from the vitreous and sclera in PBS and fixed in 4% PFA for 1 h, followed by dehydration in 30% sucrose overnight at 4 °C. Some retinas were serially sectioned at the thickness of 14 µm using a cryostat microtome. After incubation in blocking buffer containing 3% normal goat serum (NGS), 1% bovine serum albumin (BSA) and 0.3% Triton X-100 in PBS, pH 7.4, for 1 h, retina sections or cultured BV2 cells were incubated with GFAP (Dako, 1:500), Iba-1 (Wako, 1:500) and CD68 (Bio-rad, 1:500) antibodies in blocking buffer overnight. Afterwards, goat anti-rabbit Alexa Flour 488 (Invitrogen, 1:500), goat anti-rat Alexa Fluor 568 (Invitrogen, 1:500) were incubated for 2 h before mounting slides with Dako fluorescence mounting medium. For whole-mounted retinas, Iba-1 (Wako, 1:500) and CD68 (Bio-rad, 1:500) were co-incubated for 24 h before treatment with goat anti-rabbit Alexa Flour 488 (Invitrogen, 1:500) and goat anti-rat Alexa Fluor 568 (Invitrogen, 1:500) for 2 h. Confocal images of fluorescent specimens from retinas whole-mounts or sections and cultured BV2 cells were captured by a Zeiss LSM 800 Upright Confocal Microscope (Zeiss, USA). And Plan-Apochromat 40 x/1.3 oil-immersion or 20 x/0.8 objectives were used. For the quantification of fluorescence intensity of GFAP, three areas at 100 µm (central), 1 mm (middle) and 1.8 mm (peripheral) from the optical nerve head in each retinal section were captured and analyzed with Image J software. The mean density per unit area in each micrograph was calculated after setting the automatic threshold for the specific signal, as previously described [57, 58]. For quantification of microglial cells, four sampling areas with 638.9 µm × 638.9 µm squares along the dorsal–ventral axis of retinal whole-mounts at 200 µm and 1 mm from the optic nerve head on both sides were photographed, and the numbers of Iba-1+ and of CD68^+^Iba-1^+^ microglial cells were manually counted.

### Terminal deoxynucleotidyl transferase (TdT)-mediated biotinylated deoxyuridine-triphosphate (dUTP)-biotin nick-end labeling (TUNEL) assay

TUNEL assay, which is widely used to detect apoptotic cells [59, 60], was performed according to the user guide (Invitrogen). In brief, retina sections were permeabilized with proteinase K for 15 min after fixation with 4% PFA at 37 °C. Next, retinas sections were incubated with TdT reaction mixture for 1 h at 37 °C, followed by incubating with TUNEL reaction cocktails containing Alexa Fluor™ 594 Picolyl Azide and 10X Click-iT™ Plus TUNEL Reaction buffer additive (Invitrogen) for 30 min at 37 °C. Following several washes with 3% BSA in PBS, the slides were incubated with DAPI for 15–20 min and mounted with Dako fluorescence mounting medium. For quantification of TUNEL-positive cells, three views in each retinal section at 100 µm (central), 1 mm (middle) and 1.8 mm (peripheral) from the optical nerve head were captured, and TUNEL-positive cells were manually counted.

### Quantitative real-time PCR

qRT-PCR was performed as our previous studies [32]. In brief, total RNA was extracted with *TransZol* Up Plus RNA Kit (TransGen, China) and reverse transcription was performed with the TransScript® First-Strand cDNA Synthesis SuperMix (TransGen, China) before performing the real-time PCR with *PerfectStart™ Green qPCR SuperMix (TransGen, China)* by QuantStudio™ 7 Flex Real-Time PCR System (Applied Biosystems™). Gene specific primers are listed in Additional file 10: Table S1.

### Electroretinographic (ERG) analysis

ERG recording was performed as previously described [32]. In brief, after dark adaption overnight, mice were anesthetized with a mixture of ketamine hydrochloride (100 mg/kg) and xylazine (20 mg/kg), and the pupils were dilated with 1% mydriacyl (Alcon). Eyes were kept moist by treating with 3% Hypromellose lubricating gel solution in both corneas before performing the ERG recording using the Celeris ERG system (Diagnosys, USA). We first performed the scotopic ERG on dark-adapted mice with different light intensities at 0.01, 0.1, 1 and 3 cd s/m^2^. After 10-min light adaptation under background intensity at 30 cd s/m^2^, photopic ERG was recorded at 3 and 10 cd s/m^2^ light intensities. Ten sweeps were acquired with each light stimulus. The distance between the baseline and the negative peak were measured as the amplitude of ERG a-wave, and the amplitude of b-wave was calculated between the bottom of the a-wave and the top of the tallest curve.

### RNA sequencing

The mouse retinas were collected from wild-type or TREM2^−/−^ mice after LPS and siTPM1 treatments. Total RNA was isolated with RNeasy Kits (QIAGEN) and quantified with Agilent 2100 Bioanalyzer (Agilent Technologies). All samples reached a quality control threshold (RIN ≥ 8.5). mRNA was isolated from the total RNA using oligo(dT)-attached magnetic beads and fragmented to synthesize first strand cDNA using random N6-primed reverse transcription, followed by a second-strand cDNA synthesis with dUTP. After end-pair, 3′ adenylated modification and adaptor ligation, PCR amplification was performed. The single strand DNA was cyclized by splint oligo and DNA ligase, followed by DNA nanoball synthesis with phi29. Bulk RNA sequencing was performed with DNBSEQ (DNBSEQ Technology) platform. After quality control, the raw data were filtered with SOAPnuke software (https://github.com/BGI-flexlab/SOAPnuke). Of which, the reads containing the adaptor or N content > 5% and low-quality reads were removed. Differentially expressed genes were analyzed by DESeq2 software, and significant difference thresholds were set as Log_2_ Fold Change > 1 and *Q*-value < 0.05. Signaling pathway analysis was performed with IPA software. Heatmap and volcano plots were generated with Graph Pad Prism software (https://www.graphpad.com/).

### DNA sequencing

To detect Rd8-associated single nucleotide deletion, we extracted genomic DNA from C57BL/6J, TREM2^−/−^, CX3CR1^CreER^ and Rosa26^iDTR^ mice, and performed DNA sequencing as described in previous studies[61]. The sequencing data are shown in Additional file 9: Fig. S9, and all animal lines in this study excluded the Rd8 mutation.

### Statistical analysis

All experiments involving imaging and quantification were repeated at least three times. Animal numbers used in each group were indicated in figure legends. Data plotting and statistical tests were performed using GraphPad Prism software version 8.0. Data are represented as mean ± SEM and analyzed with unpaired two-tailed Student’s *t* test or one-way ANOVA followed by Tukey's multiple comparisons test. In all graphs, statistical significance was described as *p < 0.05, **p < 0.01, ***p < 0.001, ****p < 0.0001; ^*#*^*p* < 0.05, ^*##*^*p* < 0.01, ^*###*^*p* < 0.001.

## Supplementary Information

**Additional file 1: Figure S1.** Validation of TPM1 overexpression or knockdown in BV2 cells after transfection with TPM1 plasmid or siTPM1. **A** mRNA level of TPM1 in BV2 cells after TPM1 plasmid transfection. Data are presented as mean ± SEM and analyzed by unpaired two-tailed Student’s *t* test or by one-way ANOVA with Tukey’s multiple comparison test (control plasmid vs. TPM1 plasmid, **p < 0.01). Three independent experiments were performed. **B**, **C** Western blot analysis (**B**) and quantification of CD68 (**C**) in BV2 cells after TPM1 transfection. **D** mRNA level of TPM1 in BV2 cells after siCTR, siTPM1-1 or siTPM1-2 transfection. **E**, **F** Western blot analysis (**E**) and quantification of TPM1 (**F**) in BV2 cells after siCTR, siTPM1-1 or siTPM1-2 transfection. Data are presented as mean ± SEM and analyzed by one-way ANOVA with Tukey’s multiple comparison test (compared to siCTR, ***p < 0.001). Three independent experiments were performed. **G**, **H** Western blot analysis (**G**) and quantification of CD68 (**H**) in BV2 cells after siCTR, siTPM1-1 or siTPM1-2 transfection following LPS treatment. **I**, **J** Quantification of PKA (**I**) and CREB (**J**) protein levels in BV2 cells after transfection with siCTR, siTPM1-1 or siTPM1-2 followed by LPS and H89 treatment. At least four independent experiments were performed.

**Additional file 2: Figure S2.** TPM1 knockdown reduces inflammation via the PKA/CREB pathway in BV2 cells. **A**–**F** qPCR analysis of TPM1, TNF-α, IL-1β, IL-6, COX-2 and iNOS in BV2 cells following treatments with LPS and siTPM1-1, siTPM1-2, or siCTR. Data are presented as mean ± SEM and analyzed by one-way ANOVA with Tukey’s multiple comparison test (compared to LPS + siCTR or control, *p < 0.05, **p < 0.01, ***p < 0.001, ****p < 0.001; LPS + siTPM1-1 vs. LPS + siTPM1-2, **p < 0.01). Four independent experiments were performed. **G** Immunostaining of BV2 cells with with antibodies against Iba-1 and CD68 after treatments with LPS and siTPM1-1, siTPM1-2, or siCTR. Arrowheads show colocalization of microglial cells with CD68. The boxed regions are highly magnified at the right side. Scale bar, 20 µm. **H**–**M** Western blot analysis (**H**) and quantification of p-PKA, PKA, p-CREB, CREB and TPM1 (**I**–**M**) in BV2 cells following treatments with LPS and siTPM1-1, siTPM1-2, or siCTR. Data are presented as mean ± SEM and analyzed by one-way ANOVA with Tukey’s multiple comparison test (compared to LPS + siCTR or control, *p < 0.05, **p < 0.01 ***p < 0.001). Four independent experiments were performed. **N**–**Q** Western blot analysis (**N**) and quantification of p-PKA, p-CREB, and TPM1 (**O**–**Q**) in BV2 cells after transfection with siTPM1-1, siTPM1-2, or siCTR followed by LPS and H89 treatment. Data are presented as mean ± SEM and analyzed by one-way ANOVA with Tukey’s multiple comparison test (compared to LPS + siCTR or control, *p < 0.05, **p < 0.01, ***p < 0.001, ****p < 0.001; compared to LPS + siTPM1-1/siTPM1-2, *p < 0.05, **p < 0.01). Five independent experiments were performed.

**Additional file 3: Figure S3.** TPM1 knockdown reduces LPS-induced inflammation and function decline in C57BL/6J mice. **A**, **B** mRNA level of TPM1 (**A**) and IL-10 (**B**) in C57BL/6 J mouse retinas after treatments with LPS and siCTR or siTPM1-1. Data are presented as mean ± SEM and analyzed by one-way ANOVA with Tukey’s multiple comparison test (compared to LPS + siCTR or LPS + siTPM1-1, *p < 0.05, **p < 0.01). *n* = 4 or 5 mice in each group. **C**–**E** Retinal whole-mounts stained with Iba-1 and CD68 antibodies (**C**) and quantification of numbers of Iba-1^+^ (**D**) and of Iba-1^+^CD68^+^ microglial cells (**E**) in the ILP and OPL of C57BL/6J mouse retinas after treatments with LPS and siCTR or siTPM1-1. Data are presented as mean ± SEM and analyzed by one-way ANOVA with Tukey’s multiple comparison test (compared to PBS, *p < 0.05, **p < 0.01, ***p < 0.001). *n* = 3, 5, 5 mice in PBS, LPS + siCTR and LPS + siTPM1-1, respectively. **F** Quantification of fluorescence intensity of GFAP in retinal sections from C57BL/6J mice after treatments with LPS and siCTR or siTPM1-1. Data are presented as mean ± SEM and analyzed by one-way ANOVA with Tukey’s multiple comparison test (compared to LPS + siCTR, *p < 0.05, **p < 0.01). *n* = 3, 4, 4 mice in PBS, LPS + siCTR and LPS + siTPM1-1, respectively. **G**, **H** Scotopic and photopic ERG recordings on C57BL/6J mice after treatments with LPS and siCTR or siTPM1-1. Data are presented as mean ± SEM and analyzed by one-way ANOVA with Tukey’s multiple comparison test (compared to PBS, *p < 0.05). *n* = 10 mice in each group.

**Additional file 4: Figure S4.** TPM1 knockdown exacerbates inflammation in the TREM2^−/−^ mouse retina. **A**, **B** Western blot analysis (**A**) and quantification of TPM1 (**B**) in the retina of C57BL/6J (WT) and TREM2^−/−^ mice. Data are presented as mean ± SEM and analyzed by unpaired two-tailed Student’s *t* test (WT vs. TREM2^−/−^, **p < 0.01). **C**, **D** Western blot analysis (**C**) and quantification of TREM2 (**D**) in WT mice following LPS and siCTR or siTPM1-1 treatments. Data are presented as mean ± SEM and analyzed by one-way ANOVA with Tukey’s multiple comparison test (compared to PBS, **p < 0.01). *n* = 4 mice in each group (**E**) mRNA level of TPM1 in TREM2^−/−^ mice after treatments with LPS and siCTR or siTPM1-1. Data are presented as mean ± SEM and analyzed by one-way ANOVA with Tukey’s multiple comparison test (compared to LPS + siCTR, *p < 0.05). *n* = 7 mice in each group. **F** Quantification of density of microglia in the ONL of the TREM2^−/−^ mouse retina after treatments with LPS and siCTR or siTPM1-1. Data are presented as mean ± SEM and analyzed by one-way ANOVA with Tukey’s multiple comparison test (compared to PBS or LPS + siCTR, *p < 0.05, **p < 0.01). n = 4 mice in each group. **G** Quantification of fluorescence intensity of GFAP in retinal sections from TREM2^−/−^ mice after treatments with LPS and siCTR or siTPM1-1. Data are presented as mean ± SEM and analyzed by one-way ANOVA with Tukey’s multiple comparison test (compared to PBS or LPS + siCTR, *p < 0.05, **p < 0.01). *n* = 3, 4, 4 mice in PBS, LPS + siCTR and LPS + siTPM1-1, respectively. **H**, **I** Scotopic and photopic ERG recordings on TREM2^−/−^ mice after treatments with LPS and siCTR or siTPM1-1. Data are presented as mean ± SEM and analyzed by one-way ANOVA with Tukey’s multiple comparison test (compared to PBS, *p < 0.05). *n* = 13, 10, 11 mice in PBS, LPS + siCTR and LPS + siTPM1-1, respectively.

**Additional file 5: Figure S5.** TPM1 knockdown changes transcriptome associated with glial cells in WT mouse retina. **A** A heatmap showing DEGs associated with microglia and astrocytes in WT mice following treatments with PBS, or with LPS and siTPM1-1 or siCTR. **B**–**G** The expression levels of DEGs associated with M1 (**B**) or M2 microglia (**C**, **D**) and with A1 (**E**) or A2 astrocytes (**F**, **G**) in WT mice after treatment with PBS, or with LPS and siTPM1-1 or siCTR. Data are presented as mean ± SEM and analyzed by one-way ANOVA with Tukey’s multiple comparison test (compared to LPS + siCTR or LPS + siTPM1-1, *p < 0.05, **p < 0.01, ***p < 0.001, ****p < 0.001). *n* = 3 mice in each group.

**Additional file 6: Figure S6.** TPM1 knockdown elicits inflammation-related transcriptomic alterations in the WT retina. **A**, **B** DEGs associated with the phagosome formation pathway (**A**) and neuroinflammation signaling pathway (**B**) in WT mice after treatments with LPS and siTPM1-1 or siCTR.

**Additional file 7: Figure S7.** Validation of the CREB inhibitor 666-15 in TREM2^−/−^ mice following LPS treatment. **A**–**C** Western blot analysis (**A**) and quantification of p-CREB and CREB (**B**, **C**) in TREM2^−/−^ mice after treatments with LPS and 666-15, a potent and selective CREB inhibitor. Data are presented as mean ± SEM and analyzed by unpaired two-tailed Student’s *t* test (compared to LPS, ****p < 0.001). *n* = 4 mice in each group.

**Additional file 8: Figure S8.** Validation of TPM1 and TREM2 in BV2 cells after transfection with control plasmid, TPM1 plasmid, or TPM1 and Trem2 plasmids. **A** mRNA level of TPM1 in BV2 cells after transfection with control plasmid, TPM1 plasmid, or TPM1 and Trem2 plasmids. Data are presented as mean ± SEM and analyzed by one-way ANOVA with Tukey’s multiple comparison test (compared to TPM1 plasmid, **p < 0.01). Five independent experiments were performed. **B**–**D** Western blot analysis (**B**) and quantification of TREM2 (**C**) and TPM1 (**D**) in BV2 cells after transfection with control plasmid, TPM1 plasmid, or TPM1 and Trem2 plasmids. Data are presented as mean ± SEM and analyzed by one-way ANOVA with Tukey’s multiple comparison test (compared to TPM1 plasmid or TPM1 + Trem2 plasmids, *p < 0.05, **p < 0.01, ****p < 0.0001). Four independent experiments were performed.

**Additional file 9: Figure S9.** DNA sequencing for detecting Rd8-associated single nucleotide deletion. F: Forward; R: Reverse.

**Additional file 10: Table S1.** The sequences for all primers and siRNA.

## Data Availability

The datasets used and analyzed during the current study are available from the corresponding author on reasonable request.

## Abbreviations

TPM1: Tropomyosin 1
TREM2: Triggering receptor expressed on myeloid cells 2
CREB: CAMP-responsive element binding protein
LPS: Lipopolysaccharide
PKA: Protein kinase A
TLR4: Toll-like receptor 4
AD: Alzheimer’s disease
CNS: Central nervous system
NF-κB: Nuclear factor-κB
DAP12: DNAX activating protein of 12 kDa
CX3CR1: C-X3-C Motif Chemokine Receptor 1
TNF-α: Tumor necrosis factor α
IL-1β: Interleukin 1 Beta
IL-6: Interleukin 6
COX-2: Cyclooxygenase-2
iNOS: Inducible nitric oxide synthase
ONL: Outer nuclear layer
OPL: Outer plexiform layer
INL: Inner nuclear layer
IPL: Inner plexiform layer
GCL: Ganglion cell layer
NFL: Nerve fiber layer
ERG: Electroretinogram
CTR: Control
TAM: Tamoxifen
DT: Diphtheria toxin
DEGs: Differentially expressed genes
C3ar1: Complement C3a receptor 1
Ccr7: C–C Motif Chemokine Receptor 7
Itgb2: Integrin Subunit Beta 2
Ccr1: C–C Motif Chemokine Receptor 1
P2ry6: Pyrimidinergic receptor P2Y
Itgax: Integrin Subunit Alpha X
Rac2: Rac Family Small GTPase 2
Clec7a: C-Type Lectin Domain Containing 7A
Cxcl10: C-X-C motif chemokine ligand 10
Cybb: Cytochrome b and beta
Casp1: Caspase 1
Tlr13: Toll-like receptors 13
Ncf1: Neutrophil Cytosolic Factor 1
Tlr7: Toll-like receptor 7
Bax: Bcl-2 Associated X-protein
