# Supplementary figures and images for: TPM1 mediates inflammation downstream of TREM2 via the PKA/CREB signaling pathway
Source: J Neuroinflammation. 2022 Oct 14;19:257. doi: 10.1186/s12974-022-02619-3 (PMC9563125; doi:10.1186/s12974-022-02619-3)

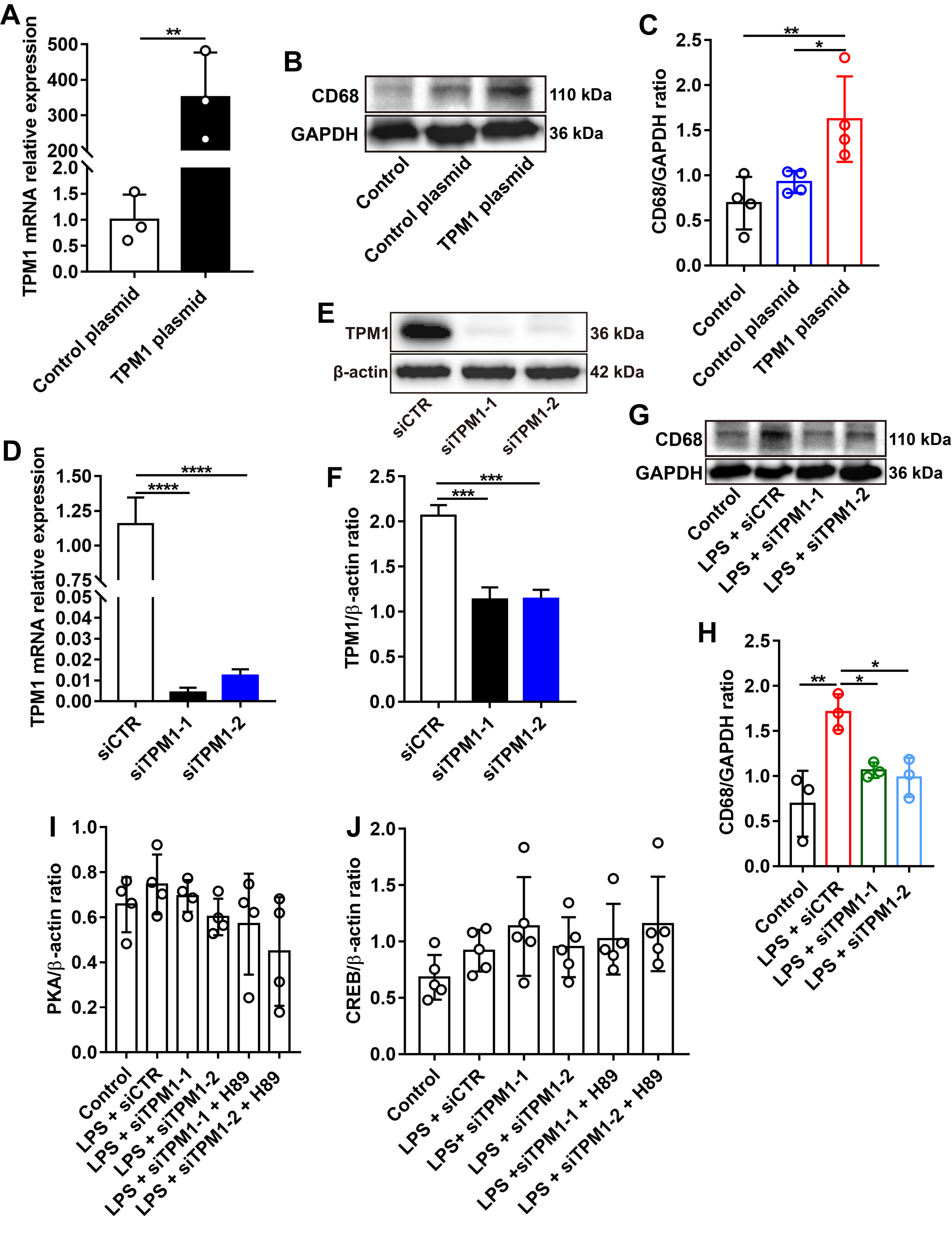

Supplement: Supplementary file 1 — Additional file 1: Figure S1. Validation of TPM1 overexpression or knockdown in BV2 cells after transfection with TPM1 plasmid or siTPM1. A mRNA level of TPM1 in BV2 cells after TPM1 plasmid transfection. Data are presented as mean ± SEM and analyzed by unpaired two-tailed Student’s t test or by one-way ANOVA with Tukey’s multiple comparison test (control plasmid vs. TPM1 plasmid, **p < 0.01). Three independent experiments were performed. B, C Western blot analysis (B) and quantification of CD68 (C) in BV2 cells after TPM1 transfection. D mRNA level of TPM1 in BV2 cells after siCTR, siTPM1-1 or siTPM1-2 transfection. E, F Western blot analysis (E) and quantification of TPM1 (F) in BV2 cells after siCTR, siTPM1-1 or siTPM1-2 transfection. Data are presented as mean ± SEM and analyzed by one-way ANOVA with Tukey’s multiple comparison test (compared to siCTR, ***p < 0.001). Three independent experiments were performed. G, H Western blot analysis (G) and quantification of CD68 (H) in BV2 cells after siCTR, siTPM1-1 or siTPM1-2 transfection following LPS treatment. I, J Quantification of PKA (I) and CREB (J) protein levels in BV2 cells after transfection with siCTR, siTPM1-1 or siTPM1-2 followed by LPS and H89 treatment. At least four independent experiments were performed. [file 12974_2022_2619_MOESM1_ESM.tif]

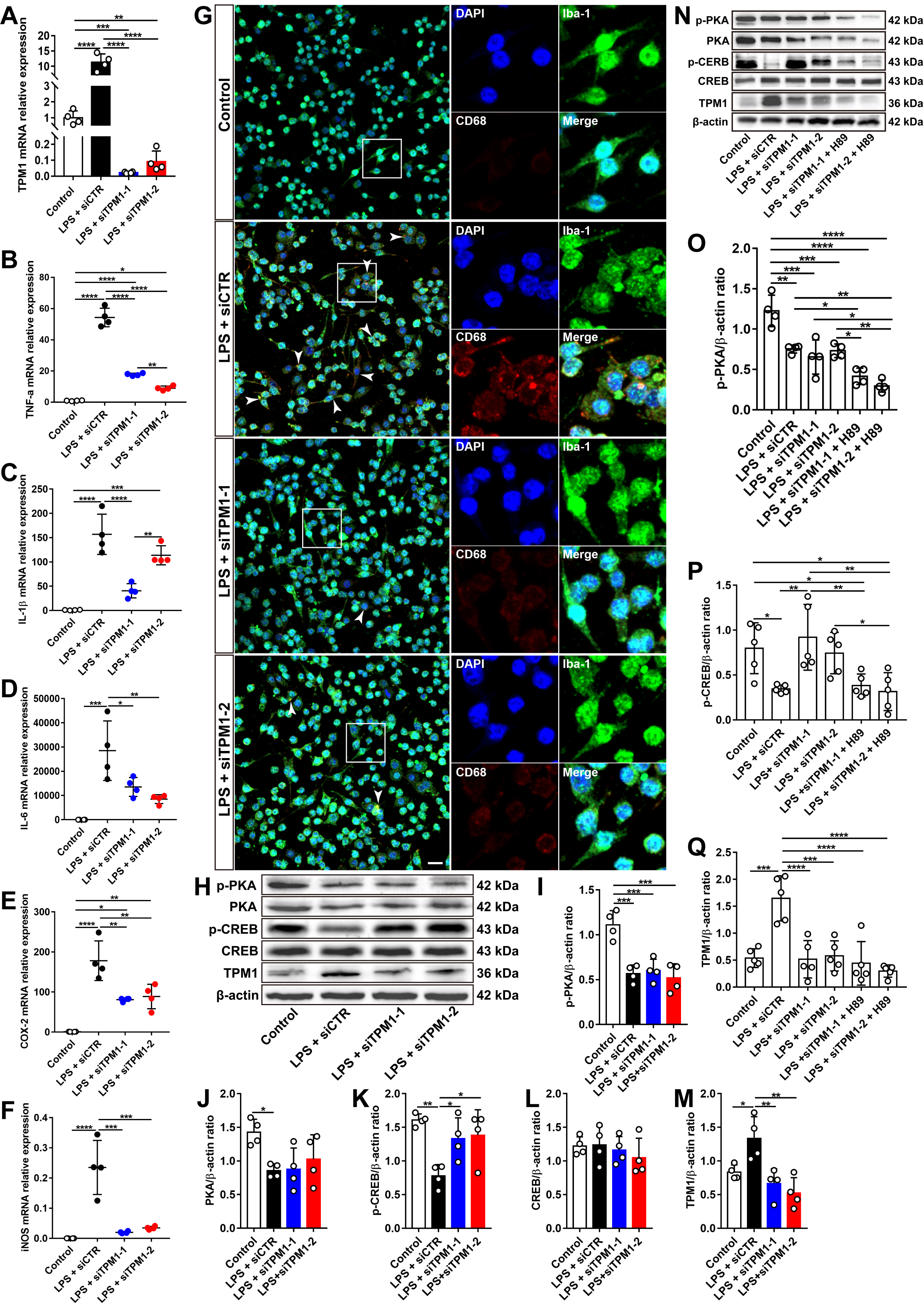

Supplement: Supplementary file 2 — Additional file 2: Figure S2. TPM1 knockdown reduces inflammation via the PKA/CREB pathway in BV2 cells. A–F qPCR analysis of TPM1, TNF-α, IL-1β, IL-6, COX-2 and iNOS in BV2 cells following treatments with LPS and siTPM1-1, siTPM1-2, or siCTR. Data are presented as mean ± SEM and analyzed by one-way ANOVA with Tukey’s multiple comparison test (compared to LPS + siCTR or control, *p < 0.05, **p < 0.01, ***p < 0.001, ****p < 0.001; LPS + siTPM1-1 vs. LPS + siTPM1-2, **p < 0.01). Four independent experiments were performed. G Immunostaining of BV2 cells with with antibodies against Iba-1 and CD68 after treatments with LPS and siTPM1-1, siTPM1-2, or siCTR. Arrowheads show colocalization of microglial cells with CD68. The boxed regions are highly magnified at the right side. Scale bar, 20 µm. H–M Western blot analysis (H) and quantification of p-PKA, PKA, p-CREB, CREB and TPM1 (I–M) in BV2 cells following treatments with LPS and siTPM1-1, siTPM1-2, or siCTR. Data are presented as mean ± SEM and analyzed by one-way ANOVA with Tukey’s multiple comparison test (compared to LPS + siCTR or control, *p < 0.05, **p < 0.01 ***p < 0.001). Four independent experiments were performed. N–Q Western blot analysis (N) and quantification of p-PKA, p-CREB, and TPM1 (O–Q) in BV2 cells after transfection with siTPM1-1, siTPM1-2, or siCTR followed by LPS and H89 treatment. Data are presented as mean ± SEM and analyzed by one-way ANOVA with Tukey’s multiple comparison test (compared to LPS + siCTR or control, *p < 0.05, **p < 0.01, ***p < 0.001, ****p < 0.001; compared to LPS + siTPM1-1/siTPM1-2, *p < 0.05, **p < 0.01). Five independent experiments were performed. [file 12974_2022_2619_MOESM2_ESM.tif]

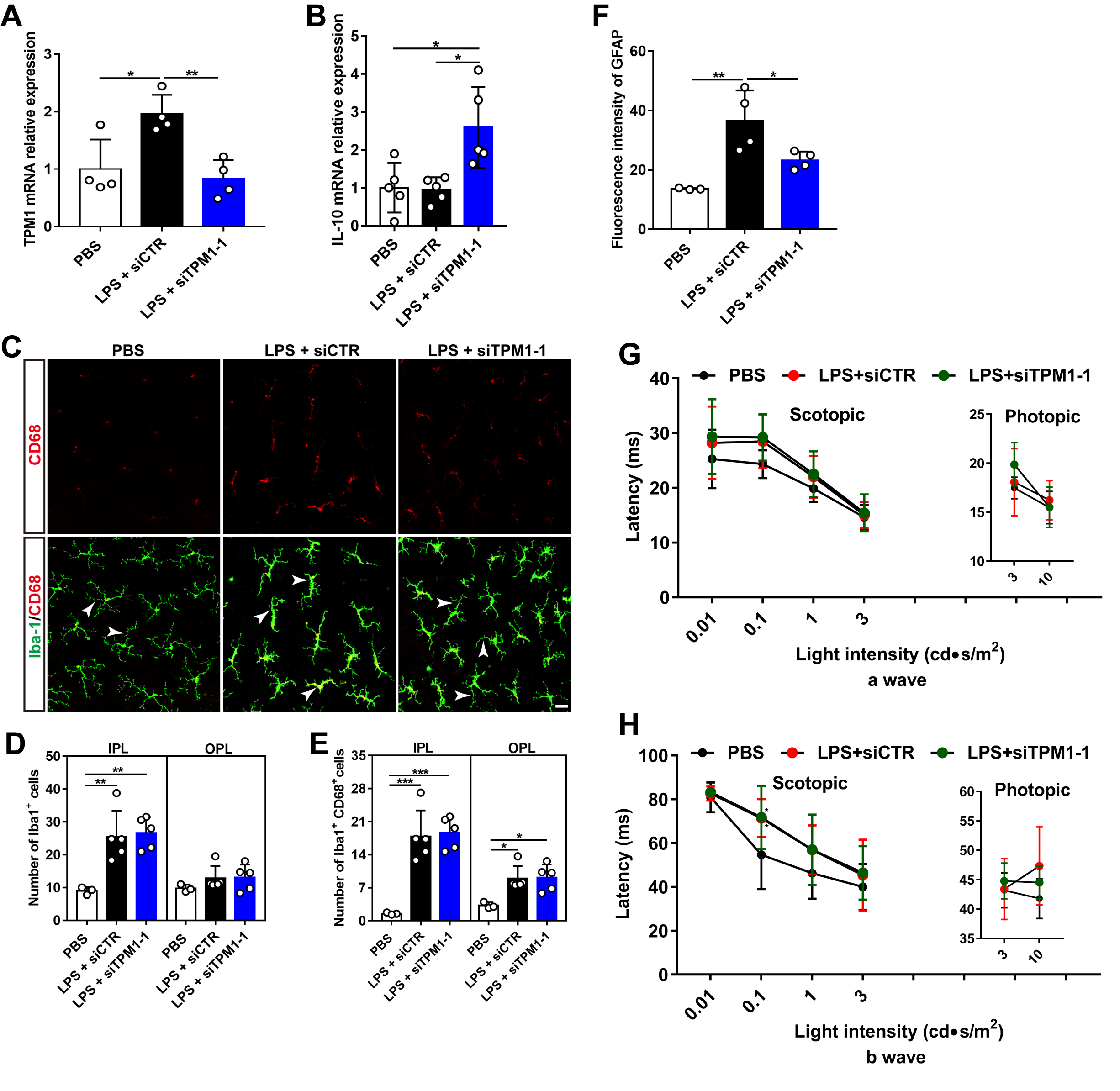

Supplement: Supplementary file 3 — Additional file 3: Figure S3. TPM1 knockdown reduces LPS-induced inflammation and function decline in C57BL/6J mice. A, B mRNA level of TPM1 (A) and IL-10 (B) in C57BL/6 J mouse retinas after treatments with LPS and siCTR or siTPM1-1. Data are presented as mean ± SEM and analyzed by one-way ANOVA with Tukey’s multiple comparison test (compared to LPS + siCTR or LPS + siTPM1-1, *p < 0.05, **p < 0.01). n = 4 or 5 mice in each group. C–E Retinal whole-mounts stained with Iba-1 and CD68 antibodies (C) and quantification of numbers of Iba-1+ (D) and of Iba-1+CD68+ microglial cells (E) in the ILP and OPL of C57BL/6J mouse retinas after treatments with LPS and siCTR or siTPM1-1. Data are presented as mean ± SEM and analyzed by one-way ANOVA with Tukey’s multiple comparison test (compared to PBS, *p < 0.05, **p < 0.01, ***p < 0.001). n = 3, 5, 5 mice in PBS, LPS + siCTR and LPS + siTPM1-1, respectively. F Quantification of fluorescence intensity of GFAP in retinal sections from C57BL/6J mice after treatments with LPS and siCTR or siTPM1-1. Data are presented as mean ± SEM and analyzed by one-way ANOVA with Tukey’s multiple comparison test (compared to LPS + siCTR, *p < 0.05, **p < 0.01). n = 3, 4, 4 mice in PBS, LPS + siCTR and LPS + siTPM1-1, respectively. G, H Scotopic and photopic ERG recordings on C57BL/6J mice after treatments with LPS and siCTR or siTPM1-1. Data are presented as mean ± SEM and analyzed by one-way ANOVA with Tukey’s multiple comparison test (compared to PBS, *p < 0.05). n = 10 mice in each group. [file 12974_2022_2619_MOESM3_ESM.tif]

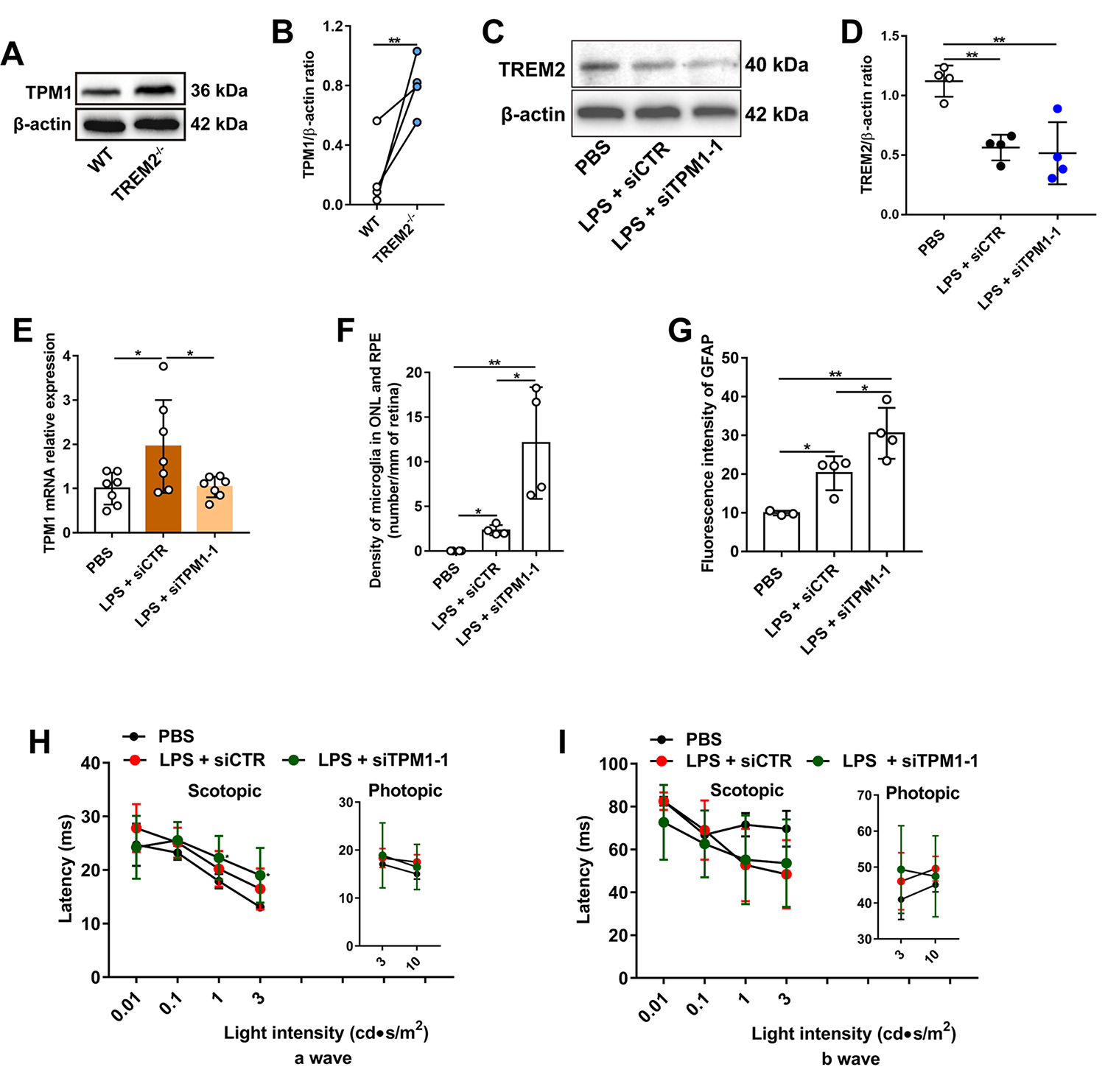

Supplement: Supplementary file 4 — Additional file 4: Figure S4. TPM1 knockdown exacerbates inflammation in the TREM2−/− mouse retina. A, B Western blot analysis (A) and quantification of TPM1 (B) in the retina of C57BL/6J (WT) and TREM2−/− mice. Data are presented as mean ± SEM and analyzed by unpaired two-tailed Student’s t test (WT vs. TREM2−/−, **p < 0.01). C, D Western blot analysis (C) and quantification of TREM2 (D) in WT mice following LPS and siCTR or siTPM1-1 treatments. Data are presented as mean ± SEM and analyzed by one-way ANOVA with Tukey’s multiple comparison test (compared to PBS, **p < 0.01). n = 4 mice in each group (E) mRNA level of TPM1 in TREM2−/− mice after treatments with LPS and siCTR or siTPM1-1. Data are presented as mean ± SEM and analyzed by one-way ANOVA with Tukey’s multiple comparison test (compared to LPS + siCTR, *p < 0.05). n = 7 mice in each group. F Quantification of density of microglia in the ONL of the TREM2−/− mouse retina after treatments with LPS and siCTR or siTPM1-1. Data are presented as mean ± SEM and analyzed by one-way ANOVA with Tukey’s multiple comparison test (compared to PBS or LPS + siCTR, *p < 0.05, **p < 0.01). n = 4 mice in each group. G Quantification of fluorescence intensity of GFAP in retinal sections from TREM2−/− mice after treatments with LPS and siCTR or siTPM1-1. Data are presented as mean ± SEM and analyzed by one-way ANOVA with Tukey’s multiple comparison test (compared to PBS or LPS + siCTR, *p < 0.05, **p < 0.01). n = 3, 4, 4 mice in PBS, LPS + siCTR and LPS + siTPM1-1, respectively. H, I Scotopic and photopic ERG recordings on TREM2−/− mice after treatments with LPS and siCTR or siTPM1-1. Data are presented as mean ± SEM and analyzed by one-way ANOVA with Tukey’s multiple comparison test (compared to PBS, *p < 0.05). n = 13, 10, 11 mice in PBS, LPS + siCTR and LPS + siTPM1-1, respectively. [file 12974_2022_2619_MOESM4_ESM.tif]

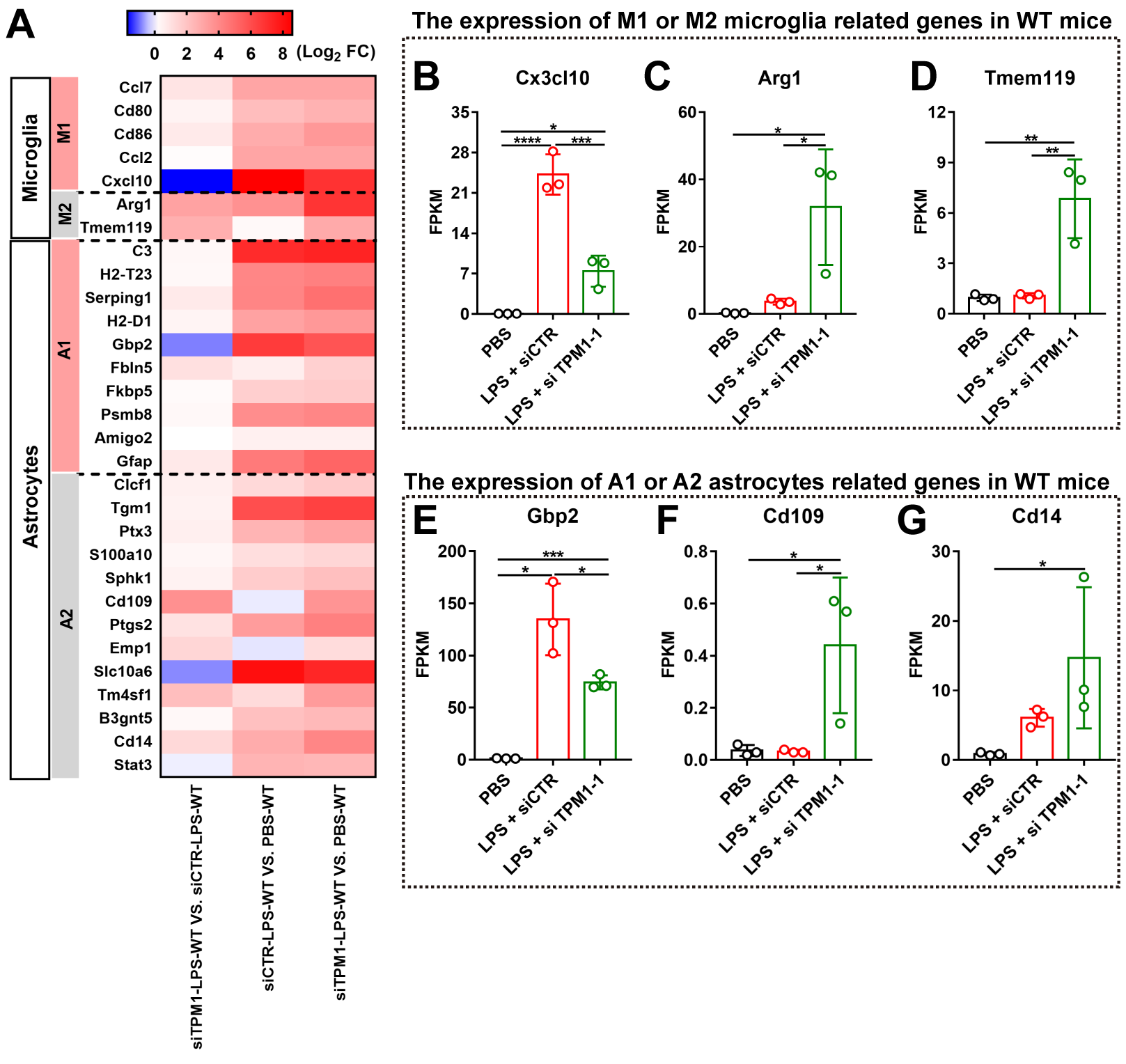

Supplement: Supplementary file 5 — Additional file 5: Figure S5. TPM1 knockdown changes transcriptome associated with glial cells in WT mouse retina. A A heatmap showing DEGs associated with microglia and astrocytes in WT mice following treatments with PBS, or with LPS and siTPM1-1 or siCTR. B–G The expression levels of DEGs associated with M1 (B) or M2 microglia (C, D) and with A1 (E) or A2 astrocytes (F, G) in WT mice after treatment with PBS, or with LPS and siTPM1-1 or siCTR. Data are presented as mean ± SEM and analyzed by one-way ANOVA with Tukey’s multiple comparison test (compared to LPS + siCTR or LPS + siTPM1-1, *p < 0.05, **p < 0.01, ***p < 0.001, ****p < 0.001). n = 3 mice in each group. [file 12974_2022_2619_MOESM5_ESM.tif]

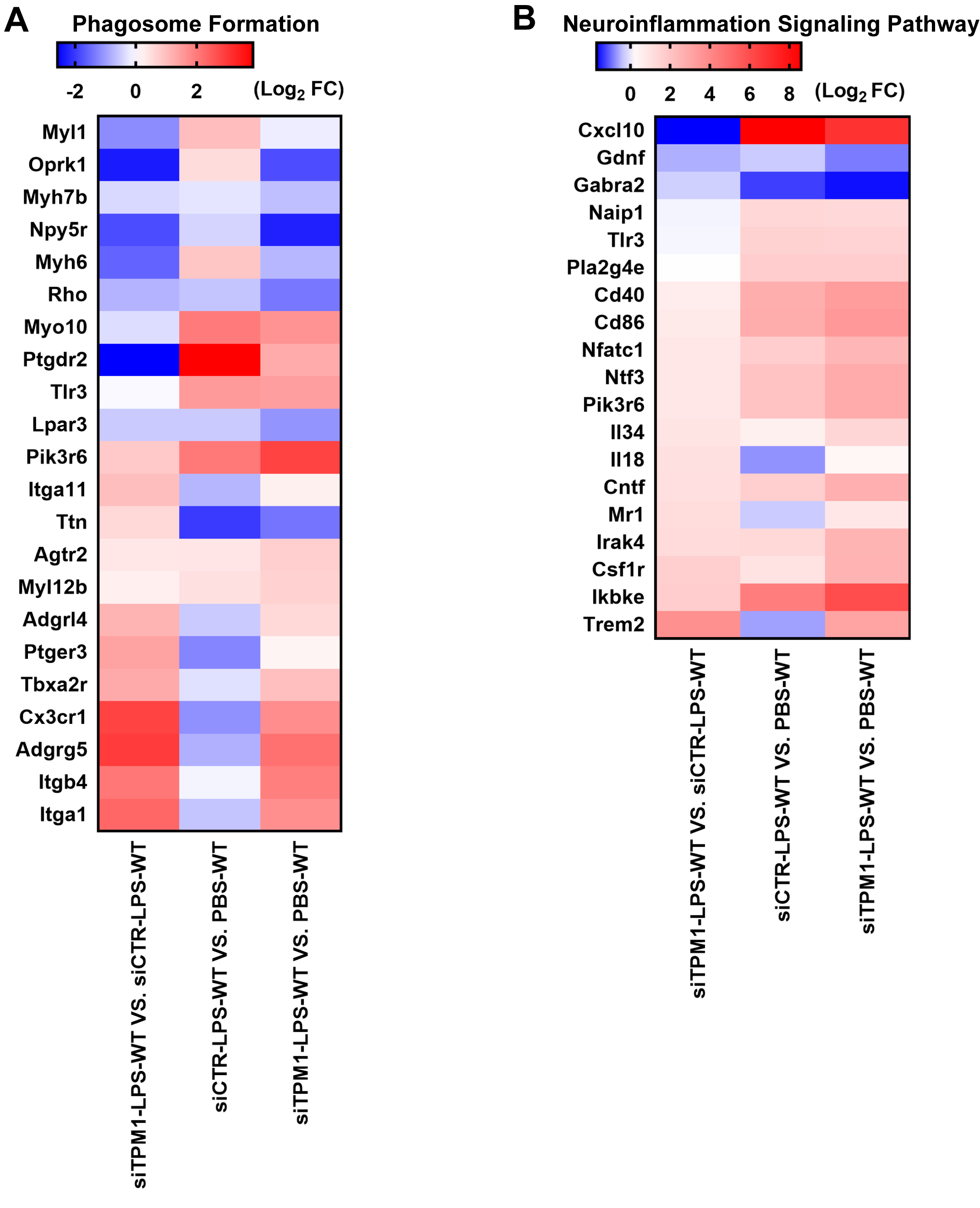

Supplement: Supplementary file 6 — Additional file 6: Figure S6. TPM1 knockdown elicits inflammation-related transcriptomic alterations in the WT retina. A, B DEGs associated with the phagosome formation pathway (A) and neuroinflammation signaling pathway (B) in WT mice after treatments with LPS and siTPM1-1 or siCTR. [file 12974_2022_2619_MOESM6_ESM.tif]

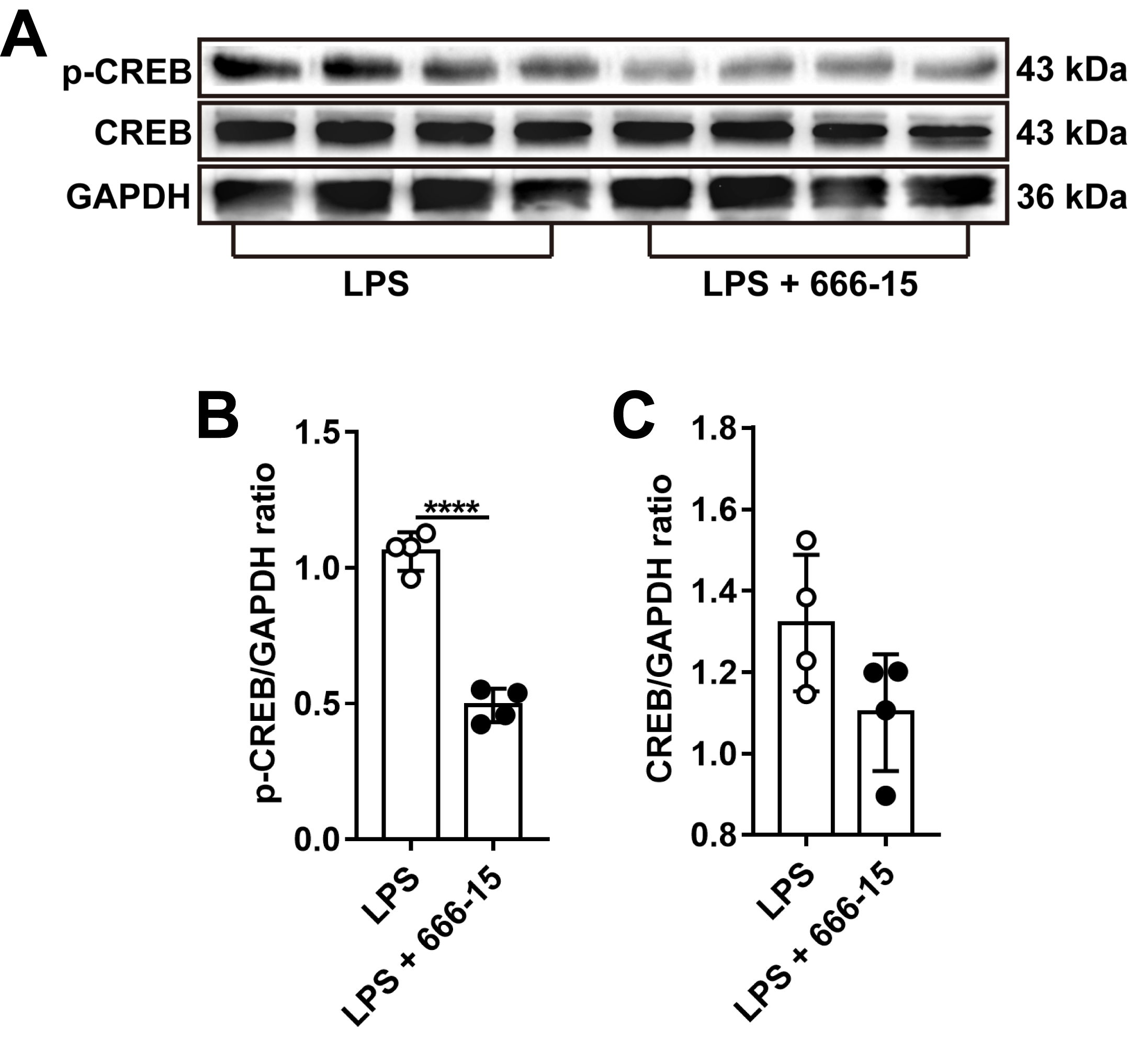

Supplement: Supplementary file 7 — Additional file 7: Figure S7. Validation of the CREB inhibitor 666-15 in TREM2−/− mice following LPS treatment. A–C Western blot analysis (A) and quantification of p-CREB and CREB (B, C) in TREM2−/− mice after treatments with LPS and 666-15, a potent and selective CREB inhibitor. Data are presented as mean ± SEM and analyzed by unpaired two-tailed Student’s t test (compared to LPS, ****p < 0.001). n = 4 mice in each group. [file 12974_2022_2619_MOESM7_ESM.tif]

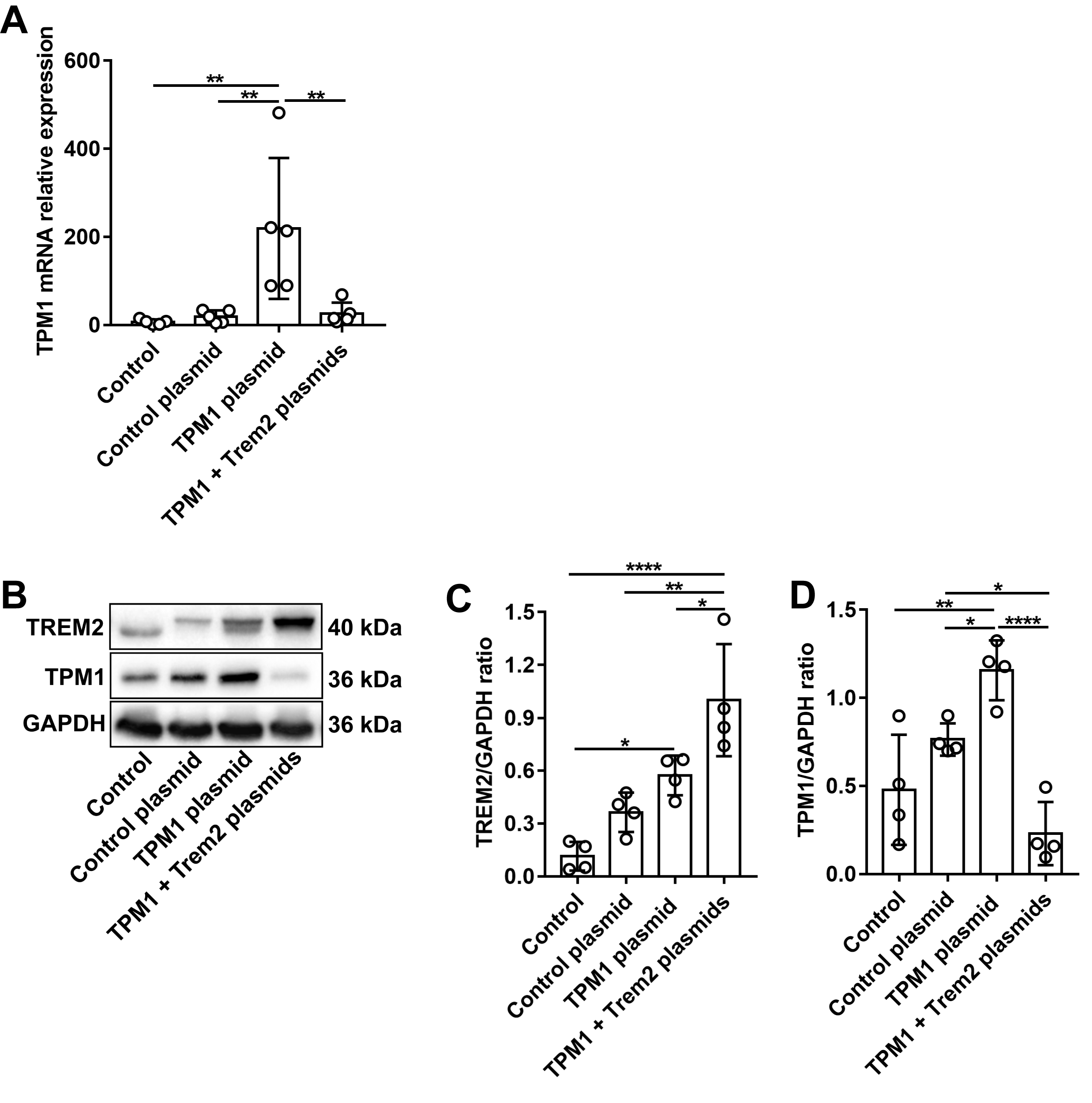

Supplement: Supplementary file 8 — Additional file 8: Figure S8. Validation of TPM1 and TREM2 in BV2 cells after transfection with control plasmid, TPM1 plasmid, or TPM1 and Trem2 plasmids. A mRNA level of TPM1 in BV2 cells after transfection with control plasmid, TPM1 plasmid, or TPM1 and Trem2 plasmids. Data are presented as mean ± SEM and analyzed by one-way ANOVA with Tukey’s multiple comparison test (compared to TPM1 plasmid, **p < 0.01). Five independent experiments were performed. B–D Western blot analysis (B) and quantification of TREM2 (C) and TPM1 (D) in BV2 cells after transfection with control plasmid, TPM1 plasmid, or TPM1 and Trem2 plasmids. Data are presented as mean ± SEM and analyzed by one-way ANOVA with Tukey’s multiple comparison test (compared to TPM1 plasmid or TPM1 + Trem2 plasmids, *p < 0.05, **p < 0.01, ****p < 0.0001). Four independent experiments were performed. [file 12974_2022_2619_MOESM8_ESM.tif]

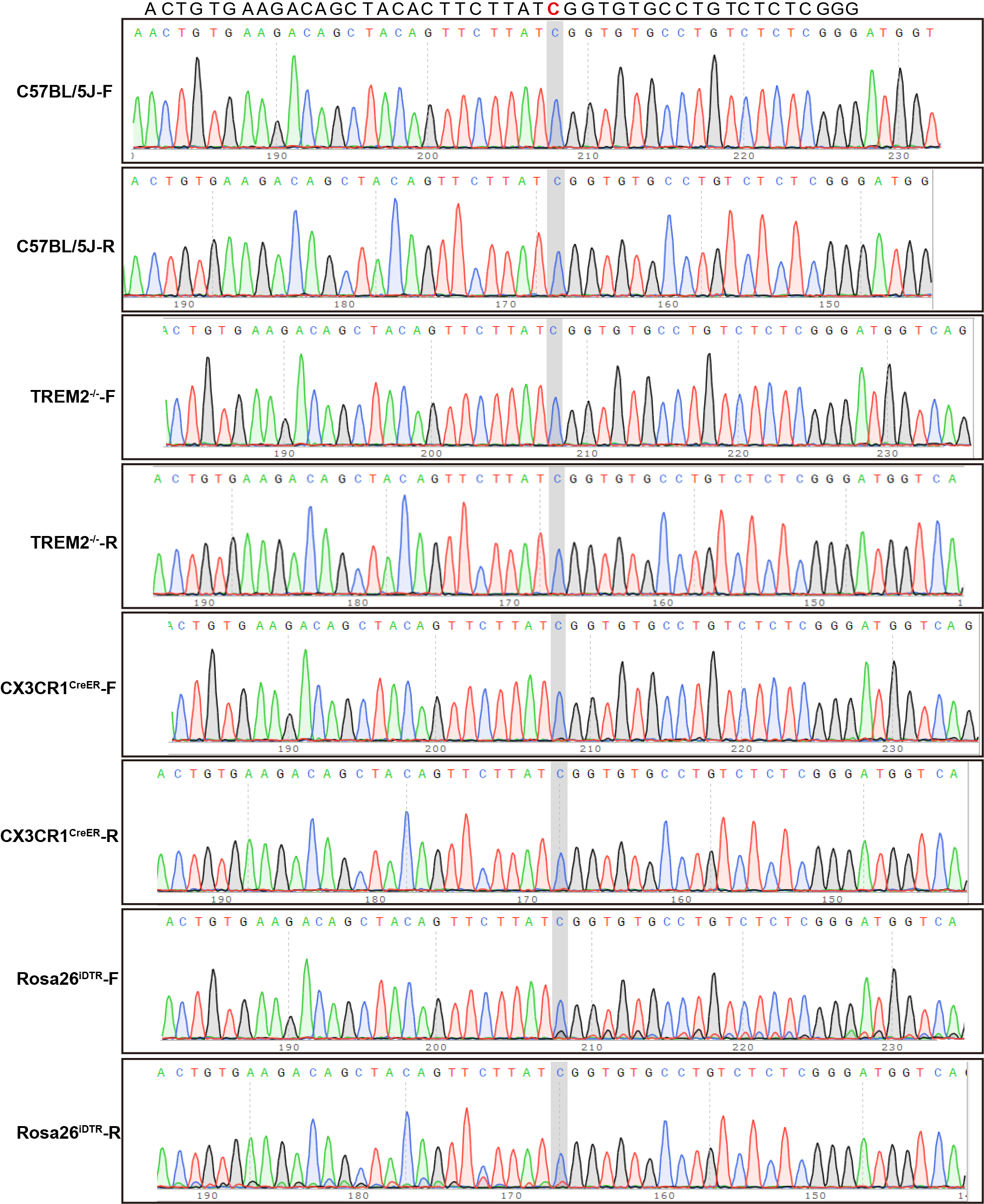

Supplement: Supplementary file 9 — Additional file 9: Figure S9. DNA sequencing for detecting Rd8-associated single nucleotide deletion. F: Forward; R: Reverse. [file 12974_2022_2619_MOESM9_ESM.tif]
